# The Endless Wars: Severe Fever With Thrombocytopenia Syndrome Virus, Host Immune and Genetic Factors

**DOI:** 10.3389/fcimb.2022.808098

**Published:** 2022-06-15

**Authors:** Min Wang, Weilong Tan, Jun Li, Liqun Fang, Ming Yue

**Affiliations:** ^1^Department of Infectious Diseases, The First Affiliated Hospital of Nanjing Medical University, Nanjing, China; ^2^Department of Infection Disease, Huadong Research Institute for Medicine and Biotechniques, Nanjing, China; ^3^State Key Lab Pathogen and Biosecurity, Beijing Institute of Microbiology and Epidemiology, Beijing, China

**Keywords:** severe fever with thrombocytopenia syndrome, viral protein, host immune response, cytokine, genetic factors

## Abstract

Severe fever with thrombocytopenia syndrome (SFTS) is an emerging arboviral infectious disease with a high rate of lethality in susceptible humans and caused by severe fever with thrombocytopenia syndrome bunyavirus (SFTSV). Currently, neither vaccine nor specific antiviral drugs are available. In recent years, given the fact that both the number of SFTS cases and epidemic regions are increasing year by year, SFTS has become a public health problem. SFTSV can be internalized into host cells through the interaction between SFTSV glycoproteins and cell receptors and can activate the host immune system to trigger antiviral immune response. However, SFTSV has evolved multiple strategies to manipulate host factors to create an optimal environment for itself. Not to be discounted, host genetic factors may be operative also in the never-ending winning or losing wars. Therefore, the identifications of SFTSV, host immune and genetic factors, and their interactions are critical for understanding the pathogenic mechanisms of SFTSV infection. This review summarizes the updated pathogenesis of SFTS with regard to virus, host immune response, and host genetic factors to provide some novel perspectives of the prevention, treatment, as well as drug and vaccine developments.

## Introduction

Severe fever with thrombocytopenia syndrome (SFTS) is an acute communicable disease caused by severe fever with thrombocytopenia syndrome bunyavirus (SFTSV), and the population is generally susceptible. The main clinical symptoms of SFTS are acute fever, gastrointestinal symptoms, myalgia, and decreased numbers of platelet and white blood cell, but they are usually self-limited. A few severe cases are accompanied by impairment of consciousness, skin petechiae, gastrointestinal and pulmonary hemorrhage, etc. Severe cases may develop multiorgan complications such as secondary encephalopathy, fulminant myocarditis, rhabdomyolysis, and hemophagocytic syndrome (Oh et al., 2016; Park et al., 2018; Zhang et al., 2018; Imataki et al., 2019; Miyamoto et al., 2019; Akagi et al., 2020). Some patients can suffer with shock, respiratory failure, and diffuse intravascular coagulation, even progress to death from multiple organ failure. SFTS was first reported in the rural areas of Hubei and Henan provinces of China in 2009 (Yu et al., 2011). In the last 10 years, the epidemic area of SFTS is expanding. Until now, SFTS has been reported in 25 provinces in China, and the number of SFTS cases has increased gradually year by year (Miao et al., 2021). Notably, it has also spread to several Southeast Asian countries. Most of the Patients with SFTS are engaged in agricultural activities in forest or hilly areas. SFTSV can be contracted through tick bites, and close contacts with animals such as cats and dogs and interpersonal dissemination have also been reported (Niu et al., 2013; Yoo et al., 2016; Jung et al., 2019b; Kobayashi et al., 2020). SFTS is mostly found from March to November and is popular during the time period between May and October, probably associated with tick activities. Currently, the fatality rate of SFTS in China is around 10%–30% (Li H. et al., 2018; Li et al., 2021; Miao et al., 2021), and there is no available vaccine or specific antiviral drugs. Given these facts, SFTS has now been one of the important infectious diseases jeopardizing public health.

Therefore, the pathogenesis of SFTS needs urgent attention. This review summarizes the latest research progress on SFTS pathogenesis from aspects including virus, host immune response, and genetic backgrounds, so as to provide references for relevant basic and clinical studies.

We searched the related references through the following electronic databases: PubMed and Web of Science. It was restricted to the English- language, literatures published during the past decade or so, with particular emphasis on the recent five years articles. The following search terms were used to retrieve the articles: “SFTS”, “SFTSV”, “severe fever with thrombocytopenia syndrome”, “severe fever with thrombocytopenia syndrome virus”, “pathogenesis”, “viral protein”, “glycoprotein”, “nucleocapsid protein”, “nonstructural protein”, “Gn”, “Gc”, “NP”, “NSs”, “immune response”, “antibody”, “cytokine”, “cytokine storm”, “process”, “outcome”, “genetic background”, “single-nucleotide polymorphism”, “SNP”, etc. In addition, all cross-references were hand-searched and all papers were screened on the basis of the relevance and coverage of their title and abstract content.

## Virus

SFTSV belongs to the order *Bunyavirales*, family *Phenuiviridae*, and genus *Phlebovirus* (Kuhn et al., 2020) and has a single, negative-strand RNA genome with three segments: large (L), medium (M), and small (S). The L segment is 6,368- nucleotide long, encoding RNA-dependent RNA polymerase (RdRp), which is responsible for genome replication and transcription (Bopp et al., 2020). The M segment comprises 3,378 nucleotides, encoding glycoprotein (Gn and Gc) precursor, which is able to mediate virus entry into the host cells (Bopp et al., 2020). The S segment includes 1,746 nucleotides, encoding nucleocapsid protein (NP) and nonstructural protein (NSs) (Bopp et al., 2020). NP serves as a carrier of genomic RNA and provides ribonucleoprotein complexes to participate in viral replication (Zhou et al., 2013), whereas NSs is a virus-encoded interferon (IFN) antagonist (Brennan et al., 2017).

### Gn/Gc Mediates Viral Invasion by Multiple Cell Receptors

Cell receptors are important “door locks” in viral entry into cells. The entry of SFTSV into the target cells requires binding of the Gn/Gc to cell receptors and fusion of viral and cell membrane, which catalyzed by conformational changes of viral proteins, followed by the release of viral RNA into the cytoplasm (Halldorsson et al., 2016; Spiegel et al., 2016; Liu et al., 2019a). In addition, the cleavage of SFTSV Gn/Gc precursors to mature Gn and Gc was found to be a prerequisite for viral entry into cells, and the process was thought to be based on the integrity of the endogenous Gc N-terminal signal peptide (Plegge et al., 2016), a low pH value (associated with the conformational change of viral protein), and the cellular serine protease activation and the reticulin participation (Hofmann et al., 2013).

Several membrane receptors were previously reported to involve in the SFTSV entry into cells. The lectin dendritic cell–specific intercellular adhesion molecule-3-grabbing non-integrin (DC-SIGN, also called CD209), for example, was considered as a responsible receptor for a large number of *Phleboviruses*, including SFTSV. It is a type of calcium-dependent lectin expressed on dendritic cells (DCs) and some tissue macrophages (Garcia-Vallejo and van Kooyk, 2013). Functionally, it can trigger immune response cascade by promoting T-lymphocyte activation and proliferation *via* the interactions with mannose or oligoses on pathogen surface (Garcia-Vallejo and van Kooyk, 2013). In addition, the expression of DC-SIGN-related protein (DC-SIGNR, also called CD209L or L-SIGN), a type-C lectin expressed on the endothelial cells of several tissues (Zhang et al., 2014), was reported to be able to enhance SFTSV invasion into the insusceptibility cells (Hofmann et al., 2013). Moreover, Sun Y. et al., 2014 found that non-muscle myosin heavy chain IIA can bind to Gn during viral infection. Non-muscle myosin heavy chain IIA, an actin-binding motor protein on cellular surfaces, was reported to induce actin cross-linking and contraction and involve in cell migration, adhesion, polarization, and morphogenesis (Vicente-Manzanares et al., 2009). It was believed to be crucial for the normal function of human platelet and vascular endothelial cell and also acts as a kind of functional viral receptor (Arii et al., 2010). When under SFTSV infection, there were several findings: the disrupted function of non-muscle myosin heavy chain IIA induced by SFTSV led to thrombocytopenia Sun Y. et al., 2014 the suppression of non-muscle myosin heavy chain IIA reduced SFTSV infection and, reciprocally, the overexpression of non-muscle myosin heavy chain IIA enhanced the cell susceptibility of SFTSV.

### Roles of NSs in SFTS Pathogenesis

#### NSs Antagonize the Generation of Interferons Through Multiple Pathways

IFNs are secreted proteins produced and released by host cells in response to viral infection and play critical roles in provoking antiviral responses. NSs was reported to be able to inhibit the exogenous IFN-α–triggered Jak/STAT signaling pathway (Chen et al., 2017) and the phosphorylation/activation of signal transducer and activator of transcription 1 (STAT1), leading to decreased IFN-stimulated genes expression and further suppressed type I/III IFN signal transduction (Chaudhary et al., 2015).

**Figure 1:**
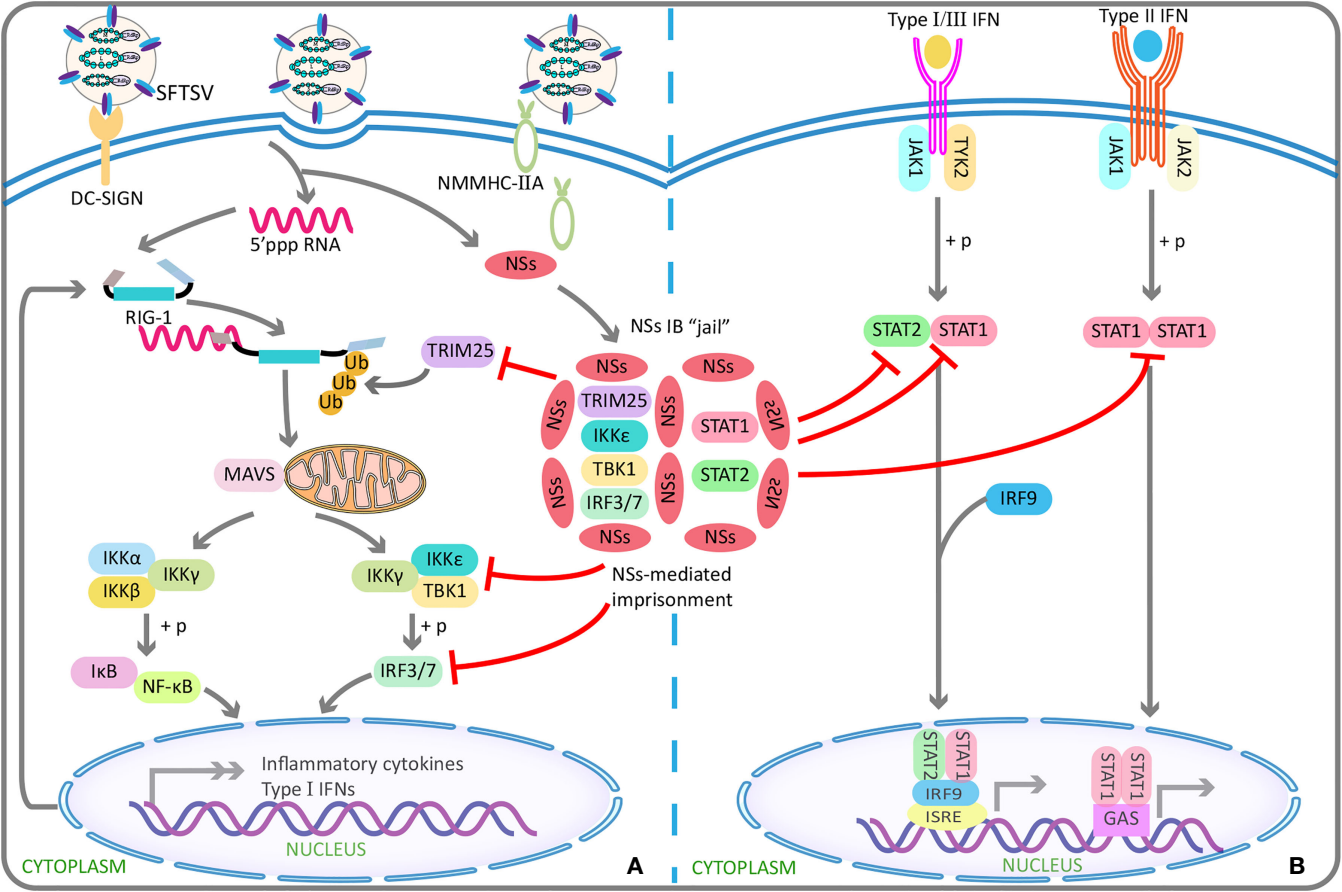
SFTSV NSs antagonizes the antiviral effect of interferons using IBs “jail”. **(A)** SFTSV NSs can capture TRIM25 into IBs in a specific manner, and subsequently inhibits TRIM25-mediated Lys-63–linked ubiquitination/activation of viral RNA sensor RIG-I, which has been found to be crucial for the RIG-I–mediated cellular antiviral response. By this indirect mechanism, the transcription and production of type I IFNs are blocked in early stage of infection. Similar to TRIM25, TBK1, IKKϵ, IRF3, and IRF7 can also be isolated in IBs by NSs, resulting in reduced production of type I IFN and finally enhanced viral replication. **(B)** SFTSV NSs can take STAT1 and STAT2 in IBs “jail”, thereby antagonizing type I/II/III IFNs signaling transduction and reducing expression of the downstream production of interferons stimulated genes involved in antiviral defense. SFTSV, severe fever with thrombocytopenia syndrome bunyavirus; NSs, nonstructural protein; IBs, inclusion bodies; TRIM25, tripartite motif 25; TBK1, TANK-binding kinase 1; IKK, IkappaB kinase; IRF, interferon regulatory factor; STAT, signal transducer and activator of transcription.

In addition, NSs can exert its anti-IFN effects *via* inducing the generation of viral inclusion bodies. That is, an *in vitro* experiment demonstrated that the viral inclusion bodies were based on lipid droplets, because NSs, lipid-coated protein A, and adipose differentiation-related protein were shown to be co-localized (Sun et al., 2016). First, in this way, by binding with TANK binding kinase 1, IkappaB kinase epsilon, IFN regulatory factor 3, and IFN regulatory factor 7, NSs can sequester them in the viral inclusion bodies, thereby inhibiting type I IFN response and finally promoting viral replication (Ning et al., 2014; Hong et al., 2019) (**Figure 1A**). The underlying mechanism may be the interaction between TANK binding kinase 1 and two conserved N-terminal amino acids of NSs (positions 21 and 23) (Moriyama et al., 2018). Second, likewise, NSs can also confine STAT1 and STAT2 into the viral inclusion bodies, thereby inhibiting the phosphorylation of STAT2 and the nuclear translocation of STAT1/2, ultimately resulting in the blocked type I IFN signaling pathway (Ning et al., 2015) (**Figure 1B**). Third, NSs can also inhibit pattern recognition receptor RIG-I–induced anti-virus type I IFN response *via* the specific capture of tripartite motif 25 into the viral inclusion bodies, followed by the subsequent suppression of tripartite motif 25–mediated RIG-I–Lys-63–linked ubiquitination and activation at the initial stage of viral infection, leading to the blocked downstream anti-virus signals (Min et al., 2020) (**Figure 1A**).

Beyond the inhibition of type I IFN–mediated antiviral innate immunity, type II IFN (IFN-γ with antiviral activity) response can also be antagonized by NSs with the involvement of the viral inclusion bodies (Ning et al., 2019). Similar to the abovementioned way, STAT1 interacted with NSs was reported to be isolated into the viral inclusion bodies, followed by the decreased STAT1 protein level, resulting in the suppression of IFN-γ production and thereby promoting SFTSV invasion (Ning et al., 2019) (**Figure 1B**). On the basis of the above studies, it seemed that the viral inclusion body is a unique structure in viral replication and immune evasion. However, the mechanism underlying the formation of viral inclusion bodies involved by NSs and the transfer to lipid droplets remain unclear.

#### NSs Show Dual-Effect in Activating and Suppressing Inflammatory Response

##### Activation of Inflammation Response

**Figure 2:**
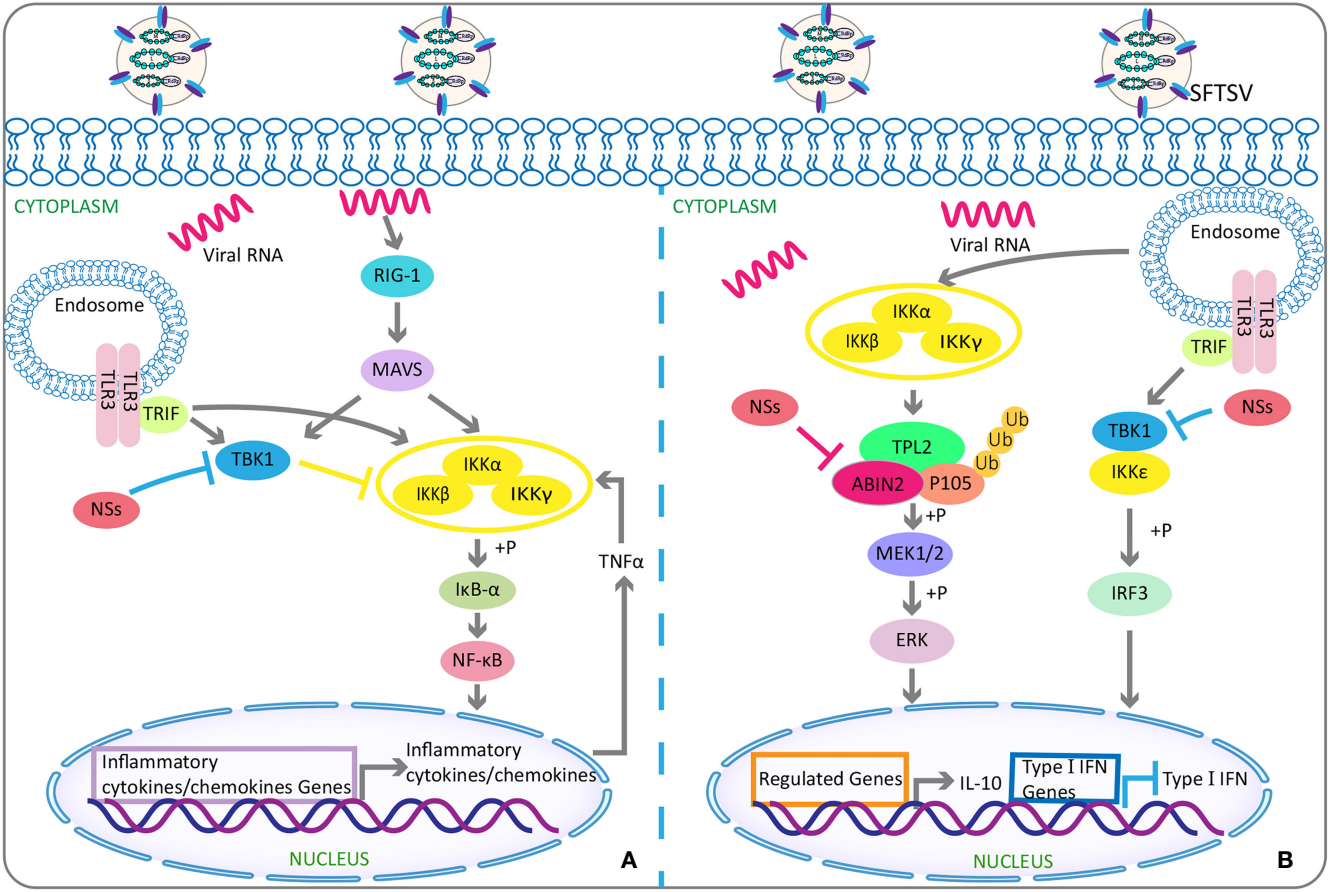
The double-edged role of SFTSV: activating or suppressing inflammation. **(A)** Activation of inflammation: Under physiological conditions, TBK1 attenuates NF-κB activity by inhibiting IKK complex. While under SFTSV-infected conditions, SFTSV NSs sequesters TBK1 into the viral inclusion bodies and then relieves the inhibitory effect of TBK1 on NF-κB signaling pathway, resulting in the activations of NF-κB and its target cytokines or chemokines genes. **(B)** Inhibition of inflammation: SFTSV NSs binds and relieves the inhibitory effect of ABIN2 on both TPL2 and p105, so as to induce the MEK/ERK signaling, finally resulting in the production of anti-inflammatory factor IL-10 for viral pathogenesis. As mentioned above, SFTSV NSs isolates TBK1 into virus inclusion bodies and eventually reduces downstream production of type I IFN. SFTSV, severe fever with thrombocytopenia syndrome bunyavirus; NSs, nonstructural protein; TBK1, TANK-binding kinase 1; NF-κB, nuclear factor-κB; IKK, IkappaB kinase; ABIN2, A20-binding inhibitor of NF-κB 2; TPL2, tumor progression locus 2; IL-10, interlukin-10.

It has been reported that NSs can activate NF-κB promoter in the epithelial cells of infected liver and enhance the target genes’ expression to trigger NF-κB–dependent inflammatory reactions (Sun Q. et al., 2015). Contrary to the abovementioned inhibitory effect of NSs on type I IFN response, NSs were observed to be able to relieve the inhibitory effect of TANK binding kinase 1 on NF-κB signaling pathway, thereby promoting the activations of NF-κB and its target cytokine or chemokine genes, but which were related to lethal cytokine storm (Khalil et al., 2020) (**Figure 2A**). These suggest that NSs and NF-κB may serve as a novel potential target for SFTS treatment.

##### Suppression of Inflammation Response

NSs can also target the tumor progression locus 2, thereby inducing the production of interleukin-10 (lL-10) (Choi et al., 2019) (**Figure 2B**), an anti-inflammatory cytokine with a significant role in suppressing immune response to avoid self-injury (Saraiva et al., 2020). Such suppression of host immune responses can be used by SFTSV as a mechanism of immune escape. In addition, NSs were found to mediate the increased expression of miR-146 in macrophages, whereas miR-146 targeted STAT1 and derived macrophages M2 polarization (Zhang L. et al., 2019). M2 macrophages were reported to reduce the production of pro-inflammatory cytokines and hence had anti-inflammatory and immune tolerance effects (Italiani and Boraschi, 2014; Wang et al., 2014), finally facilitating SFTSV replication (Zhang L. et al., 2019).

From the above, NSs might play a dual role both in activation and suppression of inflammatory response at different stages of SFTSV infection and were related to other complicated factors in the immune response. The specific roles of NSs in the pathogenesis of SFTS warrant further investigations.

#### Other Effects of NSs

**Figure 3:**
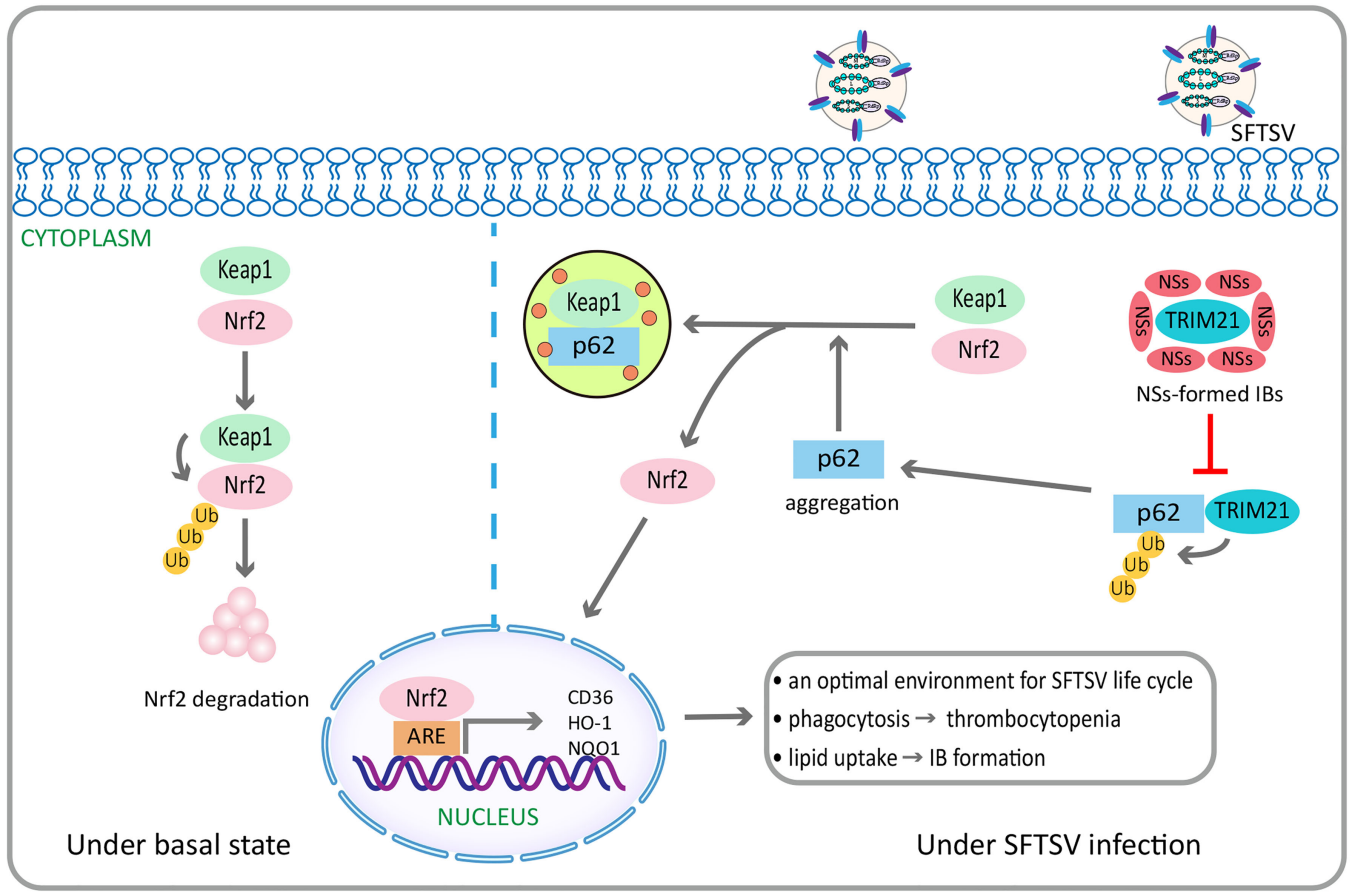
SFTSV NSs activates the p62-Keap1-Nrf2 antioxidant signal pathway through the inhibition of TRIM21, contributing to efficient pathogenesis. Under basal state, Keap1 induces Nrf2 degradation through ubiquitination. By contrast, under SFTSV infection conditions, SFTSV NSs specifically sequestrates TRIM21, which can promote p62 ubiquitination, into virus inclusion bodies, thus enhancing p62 stability and oligomerization. This promotes p62-mediated Keap1 isolation, ultimately increasing Nrf2 mediated transcriptional activation of antioxidant genes, including HO-1, NQO1, and CD36. HO-1 is endowed with anti-Inflammatory and immune-modulating properties, which can repolarizate macrophages from M1 to M2 phenotype. NQO1 is a cytoplasmic two-electron reductase responsible for the reduction of quinones to hydroquinones and the prevention of radical species production. CD36 is a scavenger receptor that can mediate lipid uptake and is regulated by Nrf2. SFTSV NSs enhances the CD36 expression through the activation of Nrf2 pathway, thereby increasing lipid uptake and CD36-mediated phagocytosis in inflammatory macrophages. It is speculated to facilitate inclusion bodies formation related to increased SFTSV replication, as well as the development of thrombocytopenia. SFTSV, severe fever with thrombocytopenia syndrome bunyavirus; NSs, nonstructural protein; TRIM21, tripartite motif 21; Keap1, Kelch-like epichlorohydrin-associated protein 1; Nrf2, nuclear factor erythroid 2–related factor 2; HO-1, heme oxygenase 1; NQO1, NAD(P)H quinone oxidoreductase 1.

NSs targeted tripartite motif 21–regulated p62-kelch–like epichlorohydrin (ECH)-associated protein 1–nuclear factor erythroid 2–related factor 2 antioxidant response signaling pathway to maintain a suitable level of oxidative stress, therefore contributing to SFTSV survival and pathogenic effects (Choi et al., 2020) (**Figure 3**). NSs increased the expression of macrophage scavenger receptor CD36 through Nuclear factor erythroid 2–related factor 2 pathway (Choi et al., 2020), thus contributing to the enhanced phagocytosis of circulating virus-bound platelets, which might be one of the possible mechanisms of thrombocytopenia in patients with SFTS (Jin et al., 2012), as well as contributing to the increased lipid uptake, which might help to the viral inclusion bodies formation and viral replication (Choi et al., 2020). In addition, NSs interacted with cyclin-dependent kinase 1 and inhibited the formation and nuclear import of the cyclin B1–cyclin–dependent kinase 1 complex, thereby leading to G2/M cell cycle arrest and enhanced viral replication (Liu et al., 2020).

## Host Immune Response

### Innate Immune Response

#### Decreased Population and Dysfunction of Monocytes

The mononuclear phagocytic system, comprising monocytes, macrophages, and DCs, is the primary target at the early stage of SFTSV infection. It was suggested that the monocytes might be the main target cells during SFTSV infection, because the absolute counts and percentages of monocytes in peripheral blood were significantly lower than those of the patients in convalescence stage or healthy controls, and such reductions were positively correlated to viral load (Peng et al., 2016). In that way, these monocytes not only were induced to cell death by the inside SFTSV replication, but also had function disorders after their surviving from SFTSV infection (Peng et al., 2016). For example, the tumor necrosis factor–α (TNF-α) production of the monocytes with LPS stimulation became impaired regardless of the increased expression of Toll-like receptor 4 on monocytes derived from patients with acute SFTS (Peng et al., 2016). Thus, the decreased population and dysfunction of monocytes in SFTS cases were associated with the disease course.

#### Dysregulated Functions of Macrophage Populations

Macrophages were also the primary target cells at the end stage SFTS infection and allowed for active SFTSV replication (Matsuno et al., 2017; Suzuki et al., 2020). Activated CD163+ M2 macrophages caused abnormal, uncontrolled inflammatory regulation by oversecreting anti-inflammatory factor IL-10 (Yamaoka et al., 2020). In addition, a phenomenon called “bystander activation” found in the patients with fatal SFTS was characterized as the significantly enhanced phagocytosis in those uninfected macrophages and consequently a systemic, large-scale host inflammatory in response to SFTSV infection (Yamaoka et al., 2020). Such abnormal activated macrophages can engulf larger numbers of erythrocytes, leukocytes, and platelets, resulting in severe thrombocytopenia, lymphocytopenia, and, finally, multi-organ dysfunction/damage (Nakano et al., 2017; Jung et al., 2019a). Obvious erythrocyte phagocytosis could be observed in the bone marrow and spleen derived from dead SFTS patients (Takahashi et al., 2014). However, erythrocytopenia could barely be observed in peripheral blood of patients with SFTS. This might be due to an alternative stress erythropoiesis. It was known in the normal case that steady-state red blood cells produced new red blood cells at a constant rate, whereas macrophages in the spleen and liver cleared aging red blood cells to balance this production (Palis, 2014; Klei et al., 2017; Seu et al., 2017). Although, in the case of inflammation caused by infection or tissue injury, the steady-state erythropoiesis was inhibited (Paulson et al., 2020a), an alternative stress erythropoiesis pathway, the bone morphogenetic protein-4–dependent stress erythropoiesis pathway, was integrated into the inflammatory response and generated a new mass of erythrocytes to maintain homeostasis until stable erythropoiesis could be restored (Bennett et al., 2019; Paulson et al., 2020a; Paulson et al., 2020b).

#### Defective Maturation of DCs

DCs are a kind of potent antigen-presenting cells and play a critical role in activating cellular and humoral immunity. High levels of circulating myeloid DCs (mDCs) were observed to be a potential protective factor for patients with SFTS (Zhang et al., 2017). Similarly, Song et al. (Song et al., 2017) also found that mDCs differentiation could be suppressed by decreased Toll-like receptor 3 expression of virus-mediated CD14^+^HLA-DR^+^ monocytes. Moreover, it had been found that the massive apoptosis in monocytes and the deficiencies in IL-4 and granulocyte macrophage colony-stimulating factor in the early stage of SFTSV infection could cause the maturation defect in mDCs, diminish the antigen presentation by mDCs, impede the differentiation from naïve T cells to T follicular helper cells, and finally resulted in failure to active specific humoral response and even a risk of immunoparalysis (Song et al., 2018).

#### Alterations in the Number and Function of NK Cells

NK cells are effector cells in innate immune system and can specifically recognize and kill virus-infected cells in the early stage of infections. A previous research found that, when compared with the patients in recovery phase or mild SFTS cases, the number of NK cells in acute phase or severe SFTS cases was significantly increased but decreased to near normal level in the recovery phase (Sun L. et al., 2014). However, in another study, NK cells showed a dramatic loss at the first week of SFTSV infection and then recovered rapidly to the normal level (Lu et al., 2015). The discrepancy seen among different studies might be attributed to the small sample size and the heterogeneity among patients with different complications or clinical outcomes. A further study revealed that, in acute phase of SFTSV infection, there was a negative association between disease severity and the cell counts and percentages of CD56^dim^CD16^+^ NK cells that were the dominant NK cell subtype in peripheral blood and could mediate the antibody-dependent cytotoxic effect (Li M. et al., 2020). In addition, CD56^dim^CD16^+^ NK cells of patients in the acute phase had the increased expressions of Ki-67 and cytotoxic effector molecule granzyme B, with relatively low expression in inhibitory NK cell receptor, suggesting the enhanced cell proliferation, activation, and effector functions of CD56^dim^CD16^+^ NK cells. Together, these findings suggested that CD56^dim^ CD16^+^ NK cells were involved in the early host defense against SFTSV infection (Li M. et al., 2020).

#### γδT Cells

γδT cells are important components of the innate immune response. The activated γδT cells can exert immunomodulatory effects and mediate inflammation by secreting many kinds of cytokines (Bonneville and Scotet, 2006). A previous study evaluated the peripheral blood γδT cell subsets of patients with SFTS and found that there was a significant increased level of Vδ1 cell and a significant decreased level of Vδ2 cell in patients with SFTS (Xu et al., 2017). In addition, such decrease of Vδ2 cell level was substantially more pronounced in severe SFTS cases, suggesting an underlying relationship between the decreased level of Vδ2 cell and unfavorable disease progression (Xu et al., 2017).

### Adaptive Immune Response

#### Humoral Immunity

Adaptive humoral immunity is mainly mediated by specific antibodies that are immunoglobulins secreted by plasma cells derived from B cells. It was reported that the absence of NP-specific IgM responses in patients with SFTS at the acute phase was associated with more adverse clinical outcomes and a greater risk of mortality (Wang et al., 2019). Moreover, the abundance of NP-specific IgM responses was negatively correlated with viral load, coagulation disorders, and liver injuries (Wang et al., 2019). As a retrospective study reported, NP-specific IgM and IgG, as well as Gn-specific IgG, were absent in the deceased patients, but specific IgM of NP and Gn was generated at the acute phase of the survived patients, followed by the generation of Gn-specific IgG (Song et al., 2018). In addition, considering a close association between NP-specific IgG antibody and virus clearance, it was believed that humoral immune response was associated with the prognosis of SFTS (Song et al., 2018). Meanwhile, B-cell subsets in patients with different clinical outcomes were analyzed in this study, and it was also found that the dysregulation of B-cell subsets, the defective maturation in plasmablasts, and the impaired antibody generation ultimately caused failure of humoral immune response (Song et al., 2018). All were key characteristics of the SFTSV death cases. Matsuno et al. (Matsuno et al., 2017) measured B220-postive B cells in spleen and lymph nodes of alpha/beta IFN receptor knockout (IFNAR^−/−^) mice and noted that Pax5-postive immature B cells were the target cells of SFTSV infection, which might contribute to severe lymphocytopenia. In addition, Suzuki et al. indicated that IgG^+^ class–switched B cells might be the major target responsible for the death of SFTSV patients by analyzing histopathological samples from 22 corpses of dead patients with SFTS (Suzuki et al., 2020). In addition, Yamaoka et al. (Yamaoka et al., 2020) believed that those IgG^+^ class–switched B cells might be defective, and could not produce functional antibodies and/or neutralizing antibodies against SFTSV. Therefore, B-cell dysfunction was likely associated with the outcomes of SFTSV infection.

#### Cellular Immunity

Adaptive cellular immunity is mediated by CD4+ and CD8+ T lymphocytes, which recognizes HLA II and HLA I molecules, respectively. Helper T cell (Th) belongs to CD4+ T lymphocyte, and cytotoxic T cell (CTL) and regulatory T cell (Treg) are subsets of CD8+ T lymphocyte. It was reported that CD3+ and CD3+CD4+ T-cell numbers in patients with SFTS with poor prognosis were significantly lower than those of healthy controls and recovery patients (Liu J. et al., 2017). Moreover, the counts and percentages of CD4+ T cell and the numbers of Th1, Th2, and Treg were evidently reduced in deaths, along with the aberrant Th1/Th2 and Th17/Treg ratios, which were associated with the severity of SFTS (Li M.M. et al., 2018b). All of the above findings suggested the failure of initiating and maintaining effective responses both in humoral and CTL immunity of mortality patients, resulting in immune suppression (Li M.M. et al., 2018b). In our previous study, three T-cell subsets (including CD69+, HLA-DR+, and CTLA4+) in SFTSV-infected patients were found to show different elevation patterns at different stages of the disease. Specifically, CD69+ T cells were elevated in the acute phase, suggestive of the role in disease onset; HLA-DR+ and CTLA4+ T cells were elevated in the recovery period of surviving patients, indicating the induction of protective immune response (Li et al., 2014). Among which, HLA-DR+ T cells could stimulate B-cell proliferation by mediating antigen-binding and signal transduction (Dendrou et al., 2018), and CTLA4, a kind of T-cell costimulatory molecule on antigen-presenting cells, could transmit an inhibitory signal to T cells (Buchbinder and Hodi, 2015). Therefore, the increased HLA-DR and CTLA4 might predict the effective infection control and recovery of patients. Thus, HLA-DR and CTLA4 might play roles in check and rebalance the lymphocytes in human body.

Research on the T-cell function in surviving patients with SFTS found that the expressions of T-lymphocyte apoptotic markers (annexin V and CD95) were upregulated in early stage of infection, and the reduction of T lymphocytes might be partly caused by Fas/FasL-mediated cell apoptosis (Li M. M. et al., 2018a). In the meantime, T-cell activation, proliferation, and function were also significantly enhanced, which participated in the initiation and maintenance of effective adaptive immune response in those surviving infections (Li M. M. et al., 2018a). A further metabolomics study found that arginine was deficient in patients with SFTS, which was probably associated with the impaired T-cell function (Li M. M. et al., 2018a). Furthermore, supplementation of arginine was helpful for accelerated virus clearance and recovery from thrombocytopenia, implying the associations of arginine catabolism pathway with platelet homeostasis and T-cell dysregulation (Li M. M. et al., 2018a). This finding might provide a new direction for SFTS clinical treatment.

Taken together, the decreased counts, abnormal proportions, and dysfunctions of T-lymphocyte subsets played important roles in the mechanism of pathogenesis of SFTS, and maintaining the immune homeostasis might provide therapeutic benefit. However, further studies are still in demand to elucidate as regards the mechanisms underlying T-lymphocyte dysregulation, the reason for the kinetics difference of T-cell proliferation, activation, and function in patients with SFTS with different clinical outcomes, as well as their correlations with the viral load.

### Cytokines

Because cytokines play an important role in the pathogenesis of SFTSV and are associated with both innate immune response and adaptive immune response, they cannot be classified into either of them, so we review cytokines separately in this section.

Cytokines are a class of small soluble molecule proteins secreted by immune and tissue cells, which play vital roles in the development, differentiation, immune response, and immunoregulation of immune cells. The cytokine-mediated inflammatory response is important in disease progression during SFTSV infection. Appropriate cytokines can activate immune cells and thus enhance immune response, whereas insufficient or excessive cytokines can cause the imbalance immune response between pro-inflammatory and anti-inflammatory. The most typical example is cytokine storm, a kind of life-threatening systemic inflammatory syndrome, characterized by elevated levels of circulating cytokines, acute systemic inflammatory symptoms, and secondary organ dysfunction, which is common in severe viral hemorrhagic fever and may lead to death (Fajgenbaum and June, 2020).

As the cytokines involved in the pathogenesis of SFTS are diverse, we summarized the cytokine expression patterns and related disorders in different disease stages. However, at present, no consensus exists on uniform criteria for disease staging of SFTS. According to the most commonly reported disease staging (Liu S. et al., 2014), we assumed the following two stages: acute phase (within 2 weeks after the onset, including fever period in the first week and multi-organ dysfunction period in the second week), and recovery phase (after 2 weeks).

#### Acute Phase (Within 2 Weeks After the Onset)

##### Correlation Between Cytokines and Clinical Courses/Outcomes of SFTSV Infection

SFTSV infection continued to progress at the first 2 weeks after the illness onset. Patients with SFTS who had previous multi-organ failure but ultimately survived were found to be self-limiting at the second week, whereas the deceased patients tended to progressively deteriorate following the experience of multi-organ failure (Liu S. et al., 2014). Thus, the period of the first 2 weeks after the onset was also regarded as an “acute phase” in many studies, and the cytokine expression patterns in acute phase were summarized in this part.

In acute phase, multiple serum cytokines significantly increased with a dynamic law of changes, which suggested that the interaction between virus and immune system significantly affected the disease course and clinical outcomes. The cytokines and chemokines of patients with SFTS with different outcomes in acute phase reported in previous studies are listed in **Table 1**.

**Table 1:** Comparison of cytokine and chemokine profiles in different disease processes or outcomes of SFTS patients in previous studies.

Year	No. of Samples	Fever Period (the First Week)	Multi-Organ Dysfunction Period (the Second Week)	Fever and Multi-Organ Dysfunction Periods (Within 2 weeks of SFTS Episode)	Convalescent Phase (After 2 Weeks)	References
2012	SFTS patients: 40 (severe cases: 9, including 6 fatal cases; non-severe cases: 31) Healthy controls: 40			SFTS patients (vs. healthy controls): elevated: TNF-α, IL-6, and RANTES; decreased: IFN-γ; no difference: TGF-β. Severe cases (vs. non-severe cases): elevated: TNF-α, IP-10, and IFN-γ; no difference: IL-6, TGF-β, and RANTES Pneumonia (vs. without pneumonia): elevated: IP-10		(Deng et al., 2012)
2012	SFTS patients: 59 (fatal cases: 15, survivors: 44) Healthy controls: 20			SFTS patients (vs. healthy controls): elevated: IL1-RA, IL-6, IL-10, G-CSF, IP-10, MCP-1, IL-1β, IL-8, MIP-1α, MIP-1β, IFN-γ, and TNF-α;decreased: PDGF-BB and RANTES. Fatal cases (vs. survivors): elevated: IL1-RA, IL-6, IL-10, G-CSF, IP-10, MCP-1, IL-1β, IL-8, MIP-1α, MIP-1β, and IFN-γ.	Survivors at convalescent period (vs. survivors at fever and multi-organ dysfunction periods): elevated: IL-1β, IL-8, MIP-1α, MIP-1β, PDGF-BB, and RANTES; decreased: IL1-RA, IL-6, IL-10, G-CSF, IP-10, and MCP-1.	(Sun et al., 2012)
2014	SFTS patients: 59 (severe cases: 11, including 7 fatal cases; non-severe cases: 48) Healthy controls: 30			SFTS patients (vs. healthy controls): elevated: TNF-α, IL-8, MIP-1α, IFN-γ, HSP70, granzyme B, VEGF, IL-2, IL-5, sICAM-1, IFN-α2, GM-CSF, and sVCAM-1;decreased: PDGF-BB, tPAI-1, and GRO. Severe cases (vs. non-severe cases): elevated: IL-15, IL-10, IL-6, IP-10, TNF-α, granzyme B, HSP70, IL-8, MIP-1α, and IFN-γ.		(Ding et al., 2014)
2014	SFTS patients: 33 (fatal cases: 4, survivors: 29) Healthy controls: 32			SFTS patients (vs. healthy controls): no difference: IL-2, IL-4, TNF-α, and IL-17A. Fatal cases (vs. survivors/healthy controls): elevated: IL-6, IL-10, and IFN-γ Survivors (vs. healthy controls): elevated: IL-10; decreased: IFN-γ.		(Li et al., 2014)
2017	SFTS patients: 50 (mild cases: 36, severe cases:14) Healthy controls: 38			SFTS patients (vs. healthy controls): elevated: TNF-α, G-CSF, IFN-γ, IFN-α, MIP-1α, IL-6, MCP-1, IL-10, IP-10; decreased: RANTES. Severe cases (vs. mild cases): elevated: G-CSF, IFN-γ, IFN-α, MIP-1α, IL-6, and IP-10; no difference: TNF-α, MCP-1, IL-10, and RANTES.		(Liu M. M. et al., 2017)
2018	SFTS patients: 33 (mild cases: 7; severe cases: 26, including 11 fatal cases) Healthy controls: 10	SFTS patients (vs. healthy controls): elevated: IL-1RA, IL-6, IL-15, IL-10, TNF-α, IFN-γ, G-CSF, eotaxin, IL-8, IP-10, MCP-1, MIP-1α, MIP-1β, and fractalkine. Fatal cases (vs. non-fatal severe cases): elevated: IL-1RA, IL-6, IL-15, IL-10, TNF-α, IFN-γ, G-CSF, IL-8, IP-10, MCP-1, and MIP-1α. Non-fatal severe patients (vs. mild cases): no difference: IL-6, IL-10, IL-15, IL-1RA, TNF-α, IFN-γ, G-CSF, eotaxin, IL-8, IP-10, MCP-1, MIP-1α, MIP-1β, and fractalkine.	Fatal cases (vs. the first week): decreased: IL-6, IL-10, eotaxin, IL-8, IP-10, MCP-1, and MIP-1β.	Fatal severe cases (vs. non-fatal severe cases): elevated: IL-8, IP-10, MCP-1, and MIP-1α. Non-fatal severe cases (vs. mild cases): no difference: eotaxin, IL-8, IP-10, MCP-1, MIP-1α, MIP-1β, and fractalkine.	Fatal severe cases (vs. the first week/the second week): decreased: IL-6, IL-10, IL-15 non-fatal severe cases (vs. the first week/the second week): elevated: PDGF. Non-fatal severe cases (vs. the first week): decreased: eotaxin, IL-8, IP-10, MCP-1, MIP-1β; elevated: RANTES. Non-fatal severe patients (vs. the second week): decreased: eotaxin, IL-8, IP-10, and MCP-1.	(Hu et al., 2018)
2018	SFTS patients: 27 (fatal cases: 10, survivors: 17) Healthy controls: 21	Fatal cases (vs. survivors): elevated: IFN-γ, IL-4, IL-10, IL-12, IL-23, and TNF-α. Survivors (vs. fatal cases): elevated: GM-CSF.	Fatal cases (vs. survivors): elevated: IFN-γ, IL-6, IL-10, and TNF-α.		Fatal cases (vs. survivors): elevated: IL-6, IL-10, and TNF-α. Survivors (vs. fatal cases): elevated: IL-1β, IL-4, and IL-12.	(Song et al., 2018)
2018	SFTS patients: 11 Healthy controls: 10			SFTS patients (vs. healthy controls): elevated: IFN-α, IL-10, MCP-1, CXCL8, IP-10, G-CSF, IL-6, and MIP-1α.	SFTS patients (vs. within 2 weeks): elevated: TNF-α, IL-1β, IL-12p40, IL-13, IL-17A, RANTES, and VEGF.	(Kwon et al., 2018)
2021	SFTS patients: 100 (mild cases: 78, severe cases: 22) Asymptomatic SFTSV-infected cases: 100 Healthy controls: 100	SFTS patients (vs. asymptomatic SFTSV-infected cases/healthy controls): elevated: IL-6, IL-10, IP-10, MCP-1, and IFN-γ; decreased: IL-8, TGF-β1, and RANTES. No difference among three groups: TNF-α; significant difference among three groups after multiple comparisons: IL-6, IL-8, MCP-1, and TGF-β1; no difference among three groups after multiple comparisons: IL-10, IP-10, MIP-1α, IFN-γ, TNF-α, and RANTES. SFTS patients (vs. asymptomatic SFTSV-infected cases): elevated: IL-6, IL-10, IP-10, MCP-1, MIP-1α, and IFN-γ; decreased: IL-8, TGF-β1, and RANTES. Healthy controls (vs. asymptomatic SFTSV-infected cases): elevated: IL-8 and MCP-1; decreased: IL-6 and TGF-β1; no difference: IL-10, IP-10, MIP-1α, IFN-γ, TNF-α, and RANTES. Asymptomatic SFTSV-infected cases (vs. SFTS patients/healthy controls): elevated: TGF-β1. Asymptomatic SFTSV-infected cases (vs. SFTS patients): elevated: RANTES. Asymptomatic SFTSV-infected cases (vs. healthy controls): no difference: RANTES. Severe cases (vs. mild cases): elevated: IL-6, IL-10, IP-10, MCP-1, IFN-γ		Mild cases: IL-8, IL-10 gradually elevated; IP-10 gradually decreased; MIP-1α no significantly fluctuated; IL-6, MCP-1, TGF-β1, and RANTES different trends.		(He et al., 2021)
2021	SFTSV^+^ patients (35 infection, 3 recovery, and 8 fatality) Healthy controls: 5			SFTSV^+^ patients (vs. healthy controls): elevated: IFN-γ, IL-6, IL-8, SIRT2, CCL3, and CXCL10. High-risk group (vs. low-risk group): elevated: IFN-γ, CASP8, IL-6, MCP-3 (CCL7), SIRT2, IL-10, CXCL9, STAMBP, CCL20, and 4EBP1. Fatality (vs. healthy controls/low-risk group/high-risk group): elevated: CCL20, TNF-α, and CX3CL1.	Recovery (vs. healthy controls): elevated: IL-8, SIRT2, MCP1, CCL4, MMP-1, TNFS14, and MCP4.	(Park et al., 2021)

SFTS, severe fever with thrombocytopenia syndrome; SFTSV, severe fever with thrombocytopenia syndrome bunyavirus; IL, interleukin; IL-1RA, IL-1 receptor antagonist; RANTES, regulated on activation and normally T-cell expressed and secreted; PDGF, platelet-derived growth factor; VEGF, vascular endothelial growth factor; TNF, tumor necrosis factor; TGF, transforming growth factor; IP-10, IFN-γ–inducible protein; G-CSF, granulocyte colony-stimulating factor; GM-CSF, granulocyte macrophage colony-stimulating factor; MCP, monocyte chemotactic protein; MIP, macrophage inflammatory protein; MMP, matrix metalloproteinase; HSP70, heat shock protein 70; eotaxin, eosinophil leukocyte chemotactic factor; sVCAM-1, soluble vascular cell adhesion molecule-1; sICAM-1, soluble intercellular adhesion molecule-1; tPAI-1, plasminogen activator inhibitor-1; GRO, growth-related oncogene; SIRT2, Sirtuin 2; CCL, CC chemokine ligand; CXCL, chemokine (C-X-C motif) ligand; STAMBP, STAM-binding protein; 4EBP1: elF4E-binding protein; CX3CL1 (fractalkine), CX3C chemokine ligand 1; TNFS14, tumor necrosis factor (TNF) superfamily member 14.

Abnormally elevated expressions of 17 inflammatory mediators were found to in patients with SFTS when compared with healthy controls, regardless of disease severity or survival status of SFTS, suggesting the roles of these inflammatory mediators in disease pathogenesis (Hu et al., 2018). Moreover, the low levels of regulated upon activation normally T cell expressed and secreted (RANTES) and platelet-derived growth factor (PDGF) were reported to be indicative of a higher risk for death (Hu et al., 2018). In addition, a previous study had revealed the reductions of PDGF-BB and RANTES in patients with SFTS when compared with healthy individuals (Sun et al., 2012). High levels of multiple cytokines, such as IL-6, IL-10, IFN-γ–inducible protein (IP-10), IFN-γ, and TNF-α, were found in acute phase of one survivor (Fujikawa et al., 2019) and of one dead (Nakamura et al., 2019). In addition, the levels of TNF-a, IL-6, IP-10, and granzyme B in the dead case remained elevated regardless repeated plasma exchange (Nakamura et al., 2019). Many studies had reported similar findings (Deng et al., 2012; Ding et al., 2014; Li et al., 2014; Liu M. M. et al., 2017; Hu et al., 2018; Song et al., 2018). These results suggested that inflammatory cytokines expression patterns were prognostic markers for clinical outcomes.

Moreover, heat shock protein 70 level in acute phase was reported as an independent risk factor for the severity of SFTSV infection (Ding et al., 2014); high levels of eotaxin/fractalkine (Hu et al., 2018) and granulocyte colony-stimulating factor (Liu M. M. et al., 2017; Hu et al., 2018) were also found to be associated with poor clinical outcomes. By contrast, higher level of granulocyte macrophage colony-stimulating factor was observed at the first week in SFTS surviving patients, and elevated serum IL-1β and IL-4 were detected in SFTS survivors accompanied by alleviation of clinical conditions (Song et al., 2018), suggesting a protective role of granulocyte macrophage colony-stimulating factor, IL-1β, and IL-4. However, a much earlier studies reported that no significant differences of IL-6, transforming growth factor-β, and RANTES in acute stage were found between critical patients with SFTS and non-critical cases (Deng et al., 2012).

A comparative analysis of many serum cytokine levels among mild and severe hospitalized patients (within 7 days of episode), asymptomatic patients, and healthy controls (He et al., 2021) is presented in **Table 1**. Among them, the levels of IL-6 and IL-8 in asymptomatic cases ranged between those of patients with SFTS and healthy controls, suggesting the roles of IL-6 and IL-8 in the self-limited virus clearance. Most recently, a study based on proteomics and single-cell transcriptomics revealed that the cohorts with different outcomes, including healthy controls, patients with SFTS (both high risk and low risk), recovery cases, and fatal cases, presented different expression patterns of serum inflammatory molecules and B-cell function–related immune molecules (Park et al., 2021). Notably, dead patients with SFTSV presented overactivated inflammatory responses resulted from the release of vast pro-inflammatory cytokines and chemokines, accompanied with the aberrant inactivation of adaptive immune responses (Park et al., 2021). Plasma B cells were speculated to act as a potential viral reservoir in fatal SFTS cases (Park et al., 2021). Thus, the cytokine/chemokines profiles aforementioned have a potential to serve as biomarkers for predicting different clinical outcomes in patients with SFTS.

Altogether, the important role of cytokines in disease progression and fatal outcome in patients with SFTS is identified. Noteworthy, IFN-α, a type I IFN with functions against SFTSV infection, was also mainly involved in cytokine storm rather than in resistance SFTSV infection, suggesting that the high level of IFN-α might fail to alleviate the symptoms, instead induce abnormal inflammatory responses and participate in cytokine storm, ultimately resulting in disease deterioration (Liu M. M. et al., 2017). Nevertheless, contradictory results existed in different studies, and the discrepancy might be attributed to the small sample size and population heterogeneity, sampling time, and experimental approaches varied across studies.

##### Correlation Between Cytokines and Viral Load

Previous studies elucidated the correlation between the levels of serum cytokines/chemokines and viral load in acute phase (**Table 2**). As is well known, in acute phase of SFTSV infection, active viral replication stimulates the release of vast cytokines and chemokines, followed by host pathological damage. Moreover, it was found that, compared with survivors, the deaths had higher average viral copy numbers in acute phase, indicating a positive relationship between the viral copy number and patient mortality (Yoshikawa et al., 2014). The adverse outcome was obviously due to the possible uncontrollable pro-inflammatory responses and subsequent significant immunopathological damage triggered by high viral load.

**Table 2:** Correlation between SFTSV load and serum levels of cytokines/chemokines in SFTS patients at acute phase (within 2 weeks).

Year	No. of Samples	Correlation between SFTSV loads and serum levels of cytokines/chemokines		References
		Positive Correlation	Negative Correlation	
2012	SFTS patients: 59 (fatal cases: 15, survivors: 44) Healthy controls: 20	IL-1RA, IL-6, IL-10, MCP-1, G-CSF, IP-10, IL-8, MIP-1α, and MIP-1β	PDGF-BB and RANTES	(Sun et al., 2012)
2014	SFTS patients: 59 (severe cases: 11, including 7 fatal cases; non-severe cases: 48) Healthy controls: 30	sIL-2RA, HSP70, IL-15, IFN-γ, and sFasl		(Ding et al., 2014)
2018	SFTS patients:11 Healthy controls: 10	IFN-α, IFN-γ, IL-10, MCP-1, CXCL8, and IP-10	RANTES and VEGF	(Kwon et al., 2018)
2021	SFTS patients:100 (mild cases: 78, severe cases: 22) Asymptomatic SFTSV-infected cases: 100 Healthy controls: 100	IL-6, IL-10, and MCP-1	RANTES	(He et al., 2021)

SFTS, severe fever with thrombocytopenia syndrome; SFTSV, severe fever with thrombocytopenia syndrome bunyavirus; IL, interleukin; IL-1RA, IL-1 receptor antagonist; MCP, monocyte chemotactic protein; G-CSF, granulocyte colony-stimulating factor; IP-10, IFN-γ–inducible protein; MIP, macrophage inflammatory protein; PDGF, platelet-derived growth factor; RANTES, regulated on activation and normally T-cell expressed and secreted; VEGF, vascular endothelial growth factor; HSP70, heat shock protein 70; sFasL, soluble Fas Ligand; CXCL, chemokine (C-X-C motif) ligand.

##### Correlation Between Cytokines and Laboratory Indices

Some studies reported the correlation between the cytokine/chemokine levels and laboratory indices in the acute phase, such as platelet count, aspartate aminotransferase, alanine aminotransferase, and lactate dehydrogenase (**Table 3**). In addition, it was observed that the proportion of low-density neutrophils in peripheral blood mononuclear cells dramatically increased in acute phase, with further transformation of low-density neutrophils from normal density neutrophils (Li et al., 2019). In the meantime, low-density neutrophils were capable of engulfing more SFTSV and secreting higher levels of IL-6, IL-8, and TNF-α, which induced the endothelial cell damage in severe cases (Li et al., 2019). All these indicated that low-density neutrophils might have pro-inflammatory actions during SFTSV infection. In the presence of multi-organ dysfunction, high viral load and abnormal serum biochemical indices were also related to disease progression (Gai Z. T. et al., 2012). Combining these findings, the production of cytokines/chemokines in acute phase of SFTSV infection was associated with abnormal laboratory indices and organ dysfunction.

**Table 3:** Correlation between cytokines and various clinical parameters and virus-specific IgG titers in SFTS patients at acute phase (within 2 weeks).

Clinical parameters	Year	No. of Samples	Correlation Between Levels of Cytokines and Various Clinical Parameters and Virus-Specific IgG Titers		References
			Positive Correlation	Negative Correlation	
White blood cells	2012	SFTS patients: 40 (severe cases: 9, including 6 fatal cases) Healthy cases: 40	IFN-γ		(Deng et al., 2012)
	2012	SFTS patients: 59 (fatal cases: 15, survivors: 44) Healthy cases: 20	IL-1β		(Sun et al., 2012)
Lymphocytes	2012	SFTS patients: 40 (severe cases: 9, including 6 fatal cases) Healthy cases: 40	IFN-γ		(Deng et al., 2012)
Platelets	2012	SFTS patients: 59 (fatal cases: 15, survivors: 44) Healthy cases: 20	PDGF-BB and RANTES	G-CSF	(Sun et al., 2012)
	2014	SFTS patients: 59 (severe cases: 11, including 7 fatal cases) Healthy cases: 30	sCD40L and PDGF-BB	IL-10, sIL-2RA and IP-10	(Ding et al., 2014)
	2018	SFTS patients: 33 (mild cases: 7, severe cases: 26, including 11 fatal cases) Healthy cases: 10	PDGF and RANTES		(Hu et al., 2018)
AST	2012	SFTS patients: 59 (fatal cases: 15, survivors: 44) Healthy cases: 20	IL-1RA, G-CSF, and IL-8		(Sun et al., 2012)
	2014	SFTS patients: 59 (severe cases: 11, including 7 fatal cases) Healthy cases: 30	IL-10, sIL-2RA, HSP70, IP-10, IL-15, IL-4, IFN-γ, and tPAI-1		(Ding et al., 2014)
ALT	2012	SFTS patients: 59 (fatal cases: 15, survivors: 44) Healthy cases: 20	G-CSF		(Sun et al., 2012)
	2014	SFTS patients: 59 (severe cases: 11, including 7 fatal cases) Healthy cases: 30	IL-10, sIL-2RA, HSP70, IP-10, IL-4, IFN-γ, and tPAI-1		(Ding et al., 2014)
BUN	2012	SFTS patients: 59 (fatal cases: 15, survivors: 44) Healthy cases: 20	IL-1RA, IL-6, IL-10, G-CSF, IP-10, MCP-1, and IL-8	PDGF-BB and RANTES	(Sun et al., 2012)
LDH	2012	SFTS patients: 59 (fatal cases: 15, survivors: 44) Healthy cases: 20	IL-1RA, IL-6, IL-10, G-CSF, IP-10, MCP-1, IL-8, and MIP-1β	PDGF-BB and RANTES	(Sun et al., 2012)
	2014	SFTS patients: 59 (severe cases: 11, including 7 fatal cases) Healthy cases: 30	IL-10, sIL-2RA, HSP70, IP-10, IL-15, IL-4, and IFN-γ	sFasl and sCD40L	(Ding et al., 2014)
CK	2012	SFTS patients: 59 (fatal cases: 15, survivors: 44) Healthy cases: 20	IL-1RA, IL-6, G-CSF, MCP-1, IL-8, and MIP-1α	PDGF-BB and RANTES	(Sun et al., 2012)
	2014	SFTS patients: 59 (severe cases: 11, including 7 fatal cases) Healthy cases: 30	IL-10, sIL-2RA, HSP70, IP-10, and IL-15		(Ding et al., 2014)
CK-MB	2012	SFTS patients: 59 (fatal cases: 15, survivors: 44) Healthy cases: 20	G-CSF, IP-10, IL-8, and MIP-1β		(Sun et al., 2012)
APTT	2012	SFTS patients: 59 (fatal cases: 15, survivors: 44) Healthy cases: 20		PDGF-BB and RANTES	(Sun et al., 2012)
IgG titers at convalescent phase	2012	SFTS patients: 59 (fatal cases: 15, survivors: 44) Healthy cases: 20	RANTES	G-CSF and IP-10	(Sun et al., 2012)

SFTS, severe fever with thrombocytopenia syndrome; AST, aspartate aminotransferase; ALT, alanine aminotransferase; BUN, blood urea nitrogen; LDH, lactate dehydrogenase; CK, creatine kinase; CK-MB, creatine kinase MB; APTT, activated partial thromboplastin time; IL, interleukin; PDGF, platelet-derived growth factor; RANTES, regulated on activation and normally T-cell expressed and secreted; G-CSF, granulocyte colony-stimulating factor; sCD40L, soluble CD40 ligand; IP-10, IFN-γ–inducible protein; IL-1RA, IL-1 receptor antagonist; HSP70, heat shock protein 70; tPAI-1, plasminogen activator inhibitor; MCP, monocyte chemotactic protein; MIP, macrophage inflammatory protein.

##### Correlation Between Cytokines and Hemophagocytic Lymphohistiocytosis

Patients with severe SFTS might experience hemophagocytic lymphohistiocytosis, which associated with cytokine storm (Lee et al., 2016; Oh et al., 2016; Nakano et al., 2017; Kaneko et al., 2018; Kim et al., 2018; Jung et al., 2019). Hemophagocytic lymphohistiocytosis was characterized by persistent activation of CTLs, NK cells, and macrophages and was often accompanied with uncontrolled abnormal secretion of inflammatory cytokines, resulting in systemic inflammatory symptoms and signs (Al-Samkari and Berliner, 2018). The high mortality rate of hemophagocytic lymphohistiocytosis that was observed up to 75% seriously threatened the life of patients with SFTS (Jung et al., 2019a), so that comprehensive examination, timely diagnosis, and prompt treatment were crucial. Further in-depth research is needed to study the underlying mechanism.

##### Correlation Between Cytokines and Encephalitis

Previous clinical investigation revealed that the incidence of encephalitis in patients with SFTS was 19.1%, and the mortality rate of patients with SFTS with encephalitis was 44.7% (Cui et al., 2015). It was also noted that the levels of serum eotaxin, IFN-γ, IL-15, IL-6, IP-10, and TNF-α were significantly elevated before clinical deterioration in the confirmed encephalitis patient, suggesting the roles of these cytokines in the development of encephalitis (Cui et al., 2015). Meanwhile, a study included eight SFTS cases with suspected encephalitis/encephalopathy reported that six cases were positive for SFTSV RNA in cerebrospinal fluid examination on days 3–7 of the disease course, along with high levels of monocyte chemotactic protein 1 (MCP-1) and IL-8 in cerebrospinal fluid when compared with those cytokines of serum (Park et al., 2018). Thus, it could be speculated that SFTSV might invade cerebrospinal fluid directly, caused elevated levels of cytokines and pathological damage in the cerebrospinal fluid in patients with SFTS (Park et al., 2018). Short-course glucocorticoids might bring benefit to the critical patients with SFTS complicating encephalopathy and left no neurologic sequelae (Nakamura et al., 2018). Thus, intervention for abnormal secretion of cytokines might be an effective management strategy in patients with SFTS with encephalopathy.

##### Correlation Between Cytokines and Cell Death

SFTSV entry into host cells can be quickly recognized by pattern recognition receptors, followed by the activation of innate and adaptive immune response, as well as the production of a series of cytokines. Such host–virus interactions induce different forms of cell death depending on the cellular context. At present, there are few correlative studies of this issue accessible; only apoptosis, pyroptosis, and autophagy have so far been reported.

Apoptosis: Apoptosis is a kind of programmed cell death, characterized by cell shrinkage, nuclear fragmentation and cytoplasmic blistering (Imre, 2020). There are two major apoptotic pathways: the extrinsic and the intrinsic pathways (Imre, 2020). The extrinsic apoptotic pathway is initiated by extracellular stress stimulation triggered by the activation of death receptors, members of TNF receptor family, including TNF receptor 1, Fas (also known as CD95), and TNF-related apoptosis-inducing ligand receptors (Zhou et al., 2017; Bertheloot et al., 2021). In contrast, the intrinsic apoptosis (also known as mitochondrial apoptosis) can be activated by a wide range of intracellular stresses such as DNA damage, oxidative stress, endoplasmic reticulum stress and so on (Zhou et al., 2017; Bertheloot et al., 2021). From an *in vitro* study, it was found that SFTSV could infect and replicate in human liver epithelial cells and significantly induce the secretion of pro-inflammatory cytokines and chemokines in infected HepG2 cells, including IFN-β, IL-6, IL-8, TNF-α, RANTES, macrophage inflammatory protein-3α, and IP-10 (Sun Q. et al., 2015). Furthermore, SFTSV-induced apoptosis in HepG2 cells was triggered by both extrinsic and intrinsic apoptotic pathways and the truncated bid (tBid) acted as a bridging initiator between the two apoptotic pathways, which might contribute to hepatic pathology in SFTSV infection (Sun Q. et al., 2015). Generally, apoptosis is critical for organism development, tissue homeostasis, immunity regulation, and elimination of virus infected cells, contributing to the overall health, but it may also be related to the pathogenic mechanism. It was reported that SFTSV-infected peripheral monocytes of patients with fatal SFTS underwent severe apoptosis even at the early stage of the infection (Song et al., 2018), which might be associated with the marked inhibition of the gene expression of IL-6 and CD40L in the monocytes (Song et al., 2017), which had been reported to possess anti-apoptotic functions against various stress inducers, including viral infection (Giri et al., 2009). As can be seen, the struggles between SFTSV and hosts exist in the process of apoptosis: SFTSV can induce or antagonize this process through multiple mechanisms, so as to create an optimal environment for viral reproduction, and even affects the clinical outcomes. First, SFTSV could mediate and utilize apoptosis of immune cells through upregulation of death receptors or their ligands on the cell surface of these infected cells (Li M. M. et al., 2018a), contributing to resist the antiviral immunity of the host. A study evaluating the function of peripheral T lymphocyte at the early stage of the disease in surviving patients with SFTS found that in parallel with the absolute counts of T-lymphocyte subsets were decreased with the significantly increased expression levels of T-lymphocyte apoptosis markers (annexin V and CD95), suggesting that apoptosis of T lymphocytes may be partly mediated by the Fas/FasL pathway, eventually impairing the antiviral immune response and contributing to disease progression (Li M. M. et al., 2018a). Second, SFTSV antagonized apoptosis of THP-1 cells by upregulating the expression of anti-apoptosis proteins (such as SOD2, BCl3, CD74, and FAM129B), thereby facilitating viral persistent infection (Qu et al., 2012; Li S. et al., 2020).

Pyroptosis: Pyroptosis (also known as highly inflammatory cell death) is one special type of regulated cell death, characterized by osmotic imbalance inside the cell and early membrane rupture of the cells, hereby allowing the release of cell contents and large number of inflammatory cytokines (Imre, 2020). Some pattern recognition receptors, such as nucleotide-binding and oligomerization, leucine-rich repeat, and pyrin domain domain–containing protein 1 (NLRP1), NLRP3, NLR family C-terminal caspase-recruitment domain domain–containing protein 4 (NLRC4), and absent in melanoma 2, recruit caspase-1 and apoptosis-associated speck-like protein containing a caspase recruitment domain to form the inflammasome and play important roles in regulating inflammatory response and pyroptosis (Sharma and Kanneganti, 2016; Swanson et al., 2019). Among these inflammasomes, NLRP3 inflammasome is the most widely characterized and the activated NLRP3 inflammasome leads to caspase-1–dependent release of the pro-inflammatory cytokines, including IL-1β and IL-18, followed by gasdermin D–mediated pyroptosis (Swanson et al., 2019). A previous study found that SFTSV infection could induce the activation of bcl-2–antigenic killer/Bcl-2–associated X protein, thereby enabling cytosolic release of oxidized the mitochondrial DNA and its subsequent binding to NLRP3 inflammasome, resulting in an excessive inflammatory response (Li S. et al., 2020). Pyroptosis has been shown to contribute to the clearance of SFTSV. A latest study reported that SFTSV infection could induce the activation of the NLRP3 inflammasome and IL-1β/IL-18 secretion, resulting in pyroptosis of SFTSV-infected human peripheral blood monocytes, thereby suppressing viral replication in peripheral blood monocytes (Li et al., 2022).

Autophagy: Autophagy includes macroautophagy, microautophagy, and chaperone-mediated autophagy, among which macroautophagy is the most intensively studied form of autophagy characterized by the formation of double membrane vesicles named autophagosomes, which engulf cytoplasmic materials and fuse with lysosome for destruction (Keller et al., 2020; Mizushima and Levine, 2020). The term autophagy usually refers to macroautophagy. Autophagy is an intracellular process of elimination of unnecessary cytoplasmic materials, such as denatured cytoplasmic proteins, destroyed organelles, and intracellular pathogens that reduces cellular stress and promotes cell survival (Keller et al., 2020). It is a highly conserved cell degradation and essential to maintain cell homeostasis, recycle damaged organelles, overcome nutrient deprivation (Mizushima and Levine, 2020). In addition, autophagy can be activated by viral infection, followed by viral selective hijacking for survival or cellular selective degradation of viral proteins, potentiation of viral antigen presentation, and participation in the regulation of inflammatory and non-inflammatory responses for prevention of viral pathogenesis (Choi et al., 2018; Liang et al., 2021). Microtubule-associated protein light chain 3 (LC3) is widely used to monitor autophagy. The conversion from soluble LC3-I protein to membrane-bound LC3-II protein is a key hallmark of autophagy (Runwal et al., 2019). SFTSV infection was found to promote the accumulation of LC3-II protein in *Vero* cells, supporting the presence of autophagy. Further, such accumulation could not be inhibited by lysosomal protease inhibitor, which suggested that the autophagy pathway was hijacked by SFTSV (Sun et al., 2018). Furthermore, a most recent study confirmed that SFTSV nucleoprotein could trigger RB1CC1/FIP200-BECN1-ATG5–dependent classical autophagy flux by disrupting the binding of BECN1 to BCL2, resulting in BECN1-dependent autophagy (Yan et al., 2021). Moreover, SFTSV exploited autophagy, which not only assembled virus particles in autophagosomes but also utilized autophagic vesicles exocytosis to promote SFTSV propagation (Yan et al., 2021).

All above findings reveal the viral and host complex strategies to take advantage of host immune response. They can intervene the host cells life cycle using different mechanisms, battle for control of “key nodes”, lead to the balance or imbalance of cell homeostasis, and thus influence the survival of virus and host cell and even associate with clinical outcomes of patients with SFTS. Therefore, greater insights into the complex regulation of cell death signaling networks during SFTSV infection could contribute to refine the clinical outcomes of the disease through appropriate clinical intervention.

##### Correlation Between Cytokines and Immunopathologic Injuries

During SFTSV infection, excessive cytokines/chemokines may induce overactivation of overexpression of immune cells, followed by immunopathology and adverse clinical outcomes. This part summarized the histological and immunopathological features and the mechanisms of various organs observed in SFTSV infection.

Vascular endothelial cell injury: Hemorrhage is a prominent manifestation of SFTSV infection. Of note, 35% patients with SFTS had bleeding symptoms (Li H. et al., 2018) and severe cases were generally expired from systemic bleeding caused by disseminated intravascular coagulation (Gai Z. T. et al., 2012). Gross pathological findings in patients with fatal SFTSV reported the subcutaneous hemorrhage in multiple sites, including anterior chest, neck, elbow, groin area, abdomen, and limbs (Hiraki et al., 2014; Uehara et al., 2016; Kaneko et al., 2018; Iwao et al., 2021), also reported hemorrhage in the gastrointestinal tract and lungs (Hiraki et al., 2014; Uehara et al., 2016; Kaneko et al., 2018; Iwao et al., 2021), as well as bilateral kidney swelling with subepithelial hemorrhage in the renal pelvis (Takahashi et al., 2014). Coagulation dysfunction caused by platelet depletion was a risk factor for bleeding. Moreover, SFTSV was found to have an ability to disrupt endothelial integrity and vascular homeostasis through various mechanisms, which was considered as a crucial factor (Xu et al., 2021). First, SFTSV could inhibit the proliferation of vascular endothelial cells and delay the endothelial cell migration and tubule formation, thus affecting angiogenesis (Xu et al., 2021). Second, high viral load could directly induce apoptosis of vascular endothelial cells (Xu et al., 2021). Third, the significant and abrupt release of multiple cytokines triggered by SFTSV could not only aggravate the hemorrhage by destabilizing the endothelial cell–cell interactions but also induce the internalization of vascular endothelium cadherin (Xu et al., 2021), which was expressed specifically in endothelial cells and was essential for maintaining endothelial barrier integrity (Lampugnani et al., 2006; Lampugnani and Elisabetta, 2007). In addition, a study on animal mouse model found the vascular leak and high pro-inflammatory cytokines levels in blood and tissues of SFTSV-infected IFNAR^−/−^ mice (Westover et al., 2019). Another animal study observed the disorganization of vascular endothelium in the lungs, the significantly damaged continuity and integrity of endothelial cells in lung sections, and the leakage of red blood cells around disintegrated pulmonary vessels, spleen, and glomerulus in SFTSV-infected HuPBL-NCG mice, indicating that SFTSV infection would impair the barrier function of vascular endothelium (Xu et al., 2021). Taken together, SFTSV possesses the ability to act directly or indirectly (through multiple cytokines) on the vascular endothelial in multiple organs such as lung, gastrointestinal tract, and kidney, which may contribute to the increased extravasation of fluid and large molecules from the intravascular to extravascular spaces, followed by severe tissue edema, hypotension, shock, and death.

Liver damage: Patients with SFTS often show symptoms of impaired liver function and the degree of hepatic impairment was correlated with the severity of the clinical course (Gai Z. T. et al., 2012; Cui et al., 2014; Nakano et al., 2017; Gong et al., 2021). An *in vitro* study found that SFTSV could replicate efficiently in HepG2 and induce significant secretion of pro-inflammatory cytokines and chemokines in HepG2 cells, such as IL-6, IL-8, TNF-α, as well as CC chemokine ligand-5, macrophage inflammatory protein-3α, and IP-10, promoting virus replication and further infection-induced apoptosis (Sun Q. et al., 2015). In animal studies, both C57/BL6 mice and humanized HuPBL-NCG mice infected with SFTSV could present with liver pathological changes, such as ballooning degeneration of hepatocytes and scattered necrosis (Jin et al., 2012; Xu et al., 2021). Moreover, an aberrant induction of pro-inflammatory cytokines and chemokines, including IL-6, IL-8, CC chemokine ligand-5, and IP-10, was also observed in the liver in SFTSV-infected C57/BL6 mice (Sun Q. et al., 2015). Similarly, the autopsy reports of patients with SFTS have noted that the livers in deceased patients showed single cell necrosis, focal necrosis, multiple lobular necrosis, mild infiltration of lymphocytes and macrophages around the portal vein and mild portal fibrosis (Hiraki et al., 2014; Takahashi et al., 2014; Uehara et al., 2016; Nakano et al., 2017; Kaneko et al., 2018), and SFTSV nucleoprotein antigen-positive atypical lymphoid cells were also detected in livers (Hiraki et al., 2014; Takahashi et al., 2014; Uehara et al., 2016; Kaneko et al., 2018; Li S. et al., 2018). Furthermore, activated macrophages with hemophagocytosis were found in the liver of some deceased patients (Uehara et al., 2016; Nakano et al., 2017). However, no certain evidence of SFTSV replication in hepatocytes was found in any of the autopsy report of patient with fatal SFTS (Hiraki et al., 2014; Uehara et al., 2016; Kaneko et al., 2018; Nakamura et al., 2019). It was contrary to the results of the *in vitro* experiment, which might be due to the different types of liver cells used among the studies, especially the significantly distinct local microenvironment. To date, no SFTSV receptors had been reported or identified in liver cells surfaces. Nonetheless, as a key, frontline immune tissue, the liver contains the largest collection of phagocytes in human body, allowing a quickly switch from immune hyporesponsiveness to a strong inflammatory response to generate efficient antiviral effect and potentially have a concurrent pathological injury to liver when the level or context of microbial products or microenvironment changes (Kubes and Jenne, 2018). Therefore, it was speculated that the liver damage in patients with SFTS might be mainly due to the pathological processes (such as hemophagocytosis, hypercytokinemia, and shock state) caused by immune response rather than the direct destroy on hepatocytes by SFTSV (Hiraki et al., 2014; Uehara et al., 2016). More research about the mechanisms is warranted.

Lung injury: It was found that around 33.7% of the patients with SFTSV infection developed pneumonia as evident on chest radiography or chest computed tomography (CT) and severe patients might die due to respiratory failure (Deng et al., 2013). The abovementioned animal research also noted the obvious peribronchiolar inflammation with bronchiolar cell structure changes and significant interstitial infiltration with perivascular cuff and extensive alveolar thickening in humanized SFTSV-infected mice (Xu et al., 2021). In addition, the increased level of chemokine IP-10 is one of the features of the viral infection related to pulmonary pathology (Lev et al., 2021). Similarly, the significantly increased serum IP-10 levels were also found in patients with pneumonia at the acute phase of SFTSV infection when compared with those of patients with SFTS (Deng et al., 2012), thus allowing for the speculation that IP-10 might play a role in the pathogenesis of SFTS-related lung injury. Furthermore, the autopsy studies of SFTS cases reported increased lung weight, alveolar hemorrhage, pulmonary edema, and diffuse alveolar injury (Hiraki et al., 2014; Uehara et al., 2016; Kaneko et al., 2018; Iwao et al., 2021), indicating acute lung injury. SFTSV nucleoprotein antigen-positive atypical lymphoid cells were also detected in the lung (Hiraki et al., 2014; Li S. et al., 2018). However, the lung parenchymal cells were negative for SFTSV nucleoprotein antigen (Hiraki et al., 2014; Uehara et al., 2016; Nakamura et al., 2019). Combined with the abovementioned findings (see the section of “Host immune response–Cytokines–Acute phase–Correlation between cytokines and clinical courses/outcomes of SFTSV infection” for details), it is speculated that the increased secretion of cytokines and chemokines induced by SFTSV and the infiltration of inflammatory cells may together mediate the immunopathological injury of the lung during SFTSV infection. One point worthy of noting was the fact that invasive fungal infections were detected in lung tissue, trachea and bronchus of a minority of patients with SFTS (Hiraki et al., 2014; Uehara et al., 2016; Iwao et al., 2021), which might be related to the inhibition of host immune response by SFTSV through several different mechanisms as follow. First, SFTSV induced the production of anti-inflammatory cytokine IL-10, which suppressed the host immune system (Choi et al., 2019), and the elevated IL-10 level in patients with SFTS was confirmed to be correlated strongly with the fatal outcomes (Yoo et al., 2021). Second, the apoptosis of monocytes in the early stage of fatal SFTSV infection diminished antigen presentation by DCs, impeded the differentiation and function of T follicular helper cells, and contributed to the failure of the SFTSV-specific humoral response (Song et al., 2018). Third, the reduction of CD3+ and CD4+ T cells induced by SFTSV infection might lead to the weakening of cellular immune responses (Liu Q. et al., 2014; Sun L. et al., 2014; Weng et al., 2014). Finally, thrombocytopenia and leukopenia could be induced by a SFTSV-related cytokine storms (Saijo, 2018). On the one hand, the reduction of neutrophils, which was the most abundant cell type among circulating white blood cells, was one of the classic risk factors of invasive pulmonary aspergillosis (Aller-Garcia et al., 2017). On the other hand, considering that fungus-induced platelets activation could inhibit fungal germination and hyphal elongation, the characteristic platelet deficiency caused by SFTSV infection would make patients more susceptible to invasive Aspergillus (Speth et al., 2013). Taken together, the various immunopathological injuries in lungs of SFTSV patients were tightly associated with SFTSV itself and the immune imbalance induced by SFTSV.

Heart dysfunction: As reported, SFTSV infection could cause reversible myocardial dysfunction (Kawaguchi et al., 2016; Miyamoto et al., 2019), and the serum IL-6 and TNF-α levels at the acute phase was significantly increased (Kawaguchi et al., 2016). However, the underlying pathogenesis remains unclear. Cardiomyocyte structural disorder with vacuolar degeneration and a positive staining of SFTSV nucleoprotein antigen in the heart with a cytoplasmic pattern was observed in a patient with SFTS died of multiple organ failure (Li S. et al., 2018). An autopsy on another patient with SFTS who died of respiratory failure observed sporadic ventricular tachycardia and right-sided heart failure at the end course of the disease, but neither histopathological manifestations of pericarditis and ischemic heart disease nor the infiltration of inflammatory cells or SFTSV-infected cardiac muscle cell was found in the decedent’s heart with the complete cardiac conduction system (Iwao et al., 2021). Furthermore, reviewing the autopsy reports of patients with SFTS between 2014 and 2021, only two studies reported that SFTSV nucleoprotein antigen-positive atypical lymphocytes were detected in the hearts (Hiraki et al., 2014; Kaneko et al., 2018), whereas no evidence of SFTSV replication in heart parenchymal cells was noted in any of the autopsy report (Hiraki et al., 2014; Uehara et al., 2016; Kaneko et al., 2018; Nakamura et al., 2019; Iwao et al., 2021). It was known that the cardiomyocytes could be damaged by virus directly or indirectly (immune responses) (Yajima, 2011), and it was reasonable to associate the multiple organ dysfunction with the cytokine storm characterized by an overwhelming and imbalanced cytokines profile during SFTSV infection (Sun et al., 2012; Liu et al., 2016). Therefore, it was speculated that SFTS-related myocardial dysfunction might be mainly due to imbalanced immune, rather than the result of direct injury by SFTSV (Kawaguchi et al., 2016; Iwao et al., 2021).

Central nervous system dysfunction: Multiple studies had confirmed found that the disturbance of consciousness during SFTSV infection was one of the risk factors for a fatal outcome (Gai Z. T. et al., 2012; Cui et al., 2014), but the exact pathogenesis of central nervous system involvement remained unknown. Much had been written about the encephalitis and encephalopathy resulting from SFTSV infection in clinical investigation. A clinical study found that the serum levels of eotaxin, IFN- γ, IL-15, IL-6, IP-10, and TNF-α in the confirmed encephalitis patients were elevated remarkably before clinical deterioration, which might be related to a potential association with the development of encephalitis (Cui et al., 2015). Another study analyzed the cerebrospinal fluid profiles during the 3–7 days of the course of disease in eight patients with SFTS with suspected acute encephalitis/encephalopathy and found that six patients were positive for SFTSV RNA in cerebrospinal fluid, and MCP-1 and IL-8 levels in cerebrospinal fluid were significantly higher than those in serum (Park et al., 2018) (see the section of “Host immune response–Cytokines–Acute phase–Correlation between cytokines and encephalitis” for details). In addition, an autopsy study on a patient with SFTS with hemophagocytic syndrome and neurological involvement reported the elevated levels of IP-10, IFN-γ, IL-8, and MCP-1 during the late stage of the disease, the focal neuronal cell degeneration in the pons, the detected hemosiderin-laden macrophages around the extended microvessels, perivascular inflammatory cells infiltration, and intravascular fibrin deposition, indicating the vascular endothelial injury with inflammation (Kaneko et al., 2018). Furthermore, SFTSV nucleoprotein antigen-positive immunoblasts were found in the vascular lumina of the various brain tissues, including the midbrain, pons, medulla, basal ganglia, cerebellum, and cerebrum (Kaneko et al., 2018). However, no evidence of direct SFTSV infection of brain parenchymal cells was found in any autopsy reports (Hiraki et al., 2014; Uehara et al., 2016; Kaneko et al., 2018; Nakamura et al., 2019; Iwao et al., 2021). Therefore, it is reasonable to speculate that the brain immunopathological injury in patients with SFTS may be caused by the increased levels of cytokines, the infiltration of inflammatory cells, and the injury of vascular endothelium induced by SFTSV.

Secondary lymphoid organs (lymph nodes and spleen) injury: It was observed that the immature B cells in the spleen and lymph nodes were the target cells of the terminal stage in SFTSV-infected IFNAR^−/−^ mouse model, and the spleen and lymph nodes showed histiocytic and necrotizing lymphadenitis lesions with pyknosis and karyorrhexis of lymphocytes (Matsuno et al., 2017). Meanwhile, a large number of pro-inflammatory cytokines, such as IL-6, MCP-1, TNF-α, IFN- γ, RANTES, and IL-1β, were found to be present at high concentrations in the serum and spleen of SFTSV-infected IFNAR^−/−^ mice (Westover et al., 2019). Such elevations of many of these cytokines were significantly related with human SFTS severity (Deng et al., 2012; Sun et al., 2012; Zhang et al., 2012; Ding et al., 2014; Liu M. M. et al., 2017). Many autopsy studies recorded the necrotizing lymphadenitis in the lymph nodes, surrounded by infiltrative lymphocytes, immunoblasts and histiocytes and a large number of nuclear fragments (Hiraki et al., 2014; Takahashi et al., 2014; Uehara et al., 2016; Kaneko et al., 2018; Nakamura et al., 2019). Moreover, the spleen showed congestion and focal hemorrhage (Li et al., 2018), as well as ischemic lesions according to several autopsy series (Uehara et al., 2016; Li S. et al., 2018). An autopsy report of 22 deceased patients with SFTS confirmed that IgG^+^ class–switched B cells and macrophages in secondary lymphoid organs such as lymph nodes and spleen were the target cells at the end stage of fatal SFTSV infection (Suzuki et al., 2020), and these IgG^+^ class–switched B cells might be defective and fail to produce functional antibodies and/or neutralizing antibodies against SFTSV, resulting in the failure of humoral immunity in lethal SFTSV infection (Yamaoka et al., 2020). Moreover, the infiltration of activated macrophage and significant hemophagocytosis were also observed in the bone marrow, spleen, and lymph nodes of some patients with SFTS (Takahashi et al., 2014; Nakano et al., 2017; Kaneko et al., 2018; Kim et al., 2018; Iwao et al., 2021), which might be one of the manifestations of hemophagocytic lymphohistiocytosis secondary to SFTS. Remarkably, hemophagocytic lymphohistiocytosis and a series of clinical diseases in patients with SFTS could be the results of hyperactivated T cells and macrophages stimulated by excessive productions of pro-inflammatory cytokines (Deng et al., 2012). Thus, it could be seen that the direct attack of SFTS, the deficient host humoral responses, and the cytokine storms might be involved in the immunopathological damages of secondary lymphoid organs during SFTSV infection.

Other organs injury: At present, only a few studies on pathological injury of other organs or tissues have been reported. Autopsies on deceased patients with SFTS noted renal ischemic lesions (Uehara et al., 2016), swollen renal tubular epithelial cells (Li S. et al., 2018), adrenal and testicular ischemic lesions (Uehara et al., 2016), gastrointestinal mucosa eroded with focal necrosis, markedly edematous and congested, severely hemorrhagic (Uehara et al., 2016), as well as pancreatic mild congestion without necrosis in the parenchyma, fat, or vessels in the pancreatic interstitium or peripancreatic space (Uehara et al., 2016). However, no evidence of SFTSV-infecting parenchymal cells of these tissues was found in any autopsy reports (Hiraki et al., 2014; Uehara et al., 2016; Kaneko et al., 2018; Nakamura et al., 2019).

To summarize the immunopathology findings of the above research, both over activation and inhibition of immune response during SFTSV infection could cause immunopathological damage, thus leading to severe disease and even fatal outcomes. Currently, no specific treatment is clinically available. Thus, maintenance of remission and preventing deterioration through inhibiting the host excessive inflammatory response (such as steroid pulse therapy or plasma exchange removing redundant cytokines/chemokines and toxins) are the primary treatment strategies, so as to help patients with SFTS to reduce the immune pathological damage and improve the prognosis. Obviously, more research studies are needed to elucidate the mechanisms in the future to eventually find a specific therapy or drug for affected patients.

#### Recovery Phase (After 2 Weeks)

As shown in **Table 1**, the level of RANTES, a cytokine known to have capabilities of recruiting and activating T cells, of patients with SFTS elevated in recovery phase (Hu et al., 2018). Correspondingly, low level of RANTES predicted a high risk of death in patients with SFTS. Moreover, RANTES along with PDGF was significantly reduced and kept at low levels during the whole disease course in deaths, both of which were positively associated with platelet count (Hu et al., 2018). Furthermore, IL-1β of survived patients was found to increase in recovery phase (Sun et al., 2012; Song et al., 2018). As a case report not listed in **Table 1**, one SFTS survivor complicating with hemophagocytic lymphohistiocytosis and encephalopathy received steroid pulse therapy on day 7 of onset for consecutive 3 days, and higher levels of serum IL-1β, IL-12p40, IL-17, and vascular endothelial growth factor were found to increase from days 23 to 43 (Fujikawa et al., 2019). Similarly, IL-1β, IL-12p40, TNF-α, IL-13, IL-17A, RANTES, and vascular endothelial growth factor were also reported to increase in recovery phase of SFTS survivor (Kwon et al., 2018). Among them, IL-13 and IL-17A could trigger immune responses specific to Th2 and Th17 cells, respectively. In addition, the levels of IL-8, Sirtuin 2, MCP-1, CC chemokine ligand 4, matrix metalloproteinase-1, TNF superfamily member 14, and MCP-4 in three SFTS convalescent cases detected after their discharge for at least 2 weeks were found to significantly higher than those of healthy controls, implying the presence of persistent inflammation despite of complete virus clearance (Park et al., 2021) (**Table 1**). It had been proven that inflammatory mediators remained at high levels at the time of death in patients who died within 3 weeks but returned to normal in survivors within 3 weeks (Hu et al., 2018). Therefore, some increased specific cytokines in recovery phase of SFTS survivors were associated with the activation and differentiation of T cells, whereas other cytokines with persistent high levels in deaths suggested dysregulated inflammatory responses and a high risk of death.

In sum, abnormal cytokine/chemokine secretion in different phase of patients with SFTS could cause organ pathological damages and dysfunction, resulting in disease progression and death outcome. Although the results of cytokine secretion patterns in the acute and recovery phase of patients with SFTS among studies were not completely consistent, unique and clinically verified cytokines patterns could be used as biomarkers for predicting disease severity or survival outcome. Moreover, blocking cytokine storm may alleviate disease severity to some extent, and the application of antiviral drugs directly targeting viral replication cycle and the management of decreasing or reversing vascular leakage may help to SFTS treatment. Appropriate timing of an intervention is particularly important.

## Host Genetic Factors

As is well known, SFTSV infection is self-limited. Only a few infected people requiring hospital station, whereas most of the infected can spontaneously clear the virus. It may be related to the difference of genetic background. In China, SFTS-epidemic area, several epidemiological studies on SFTSV-specific antibodies had been performed previously among healthy people during the epidemic season. A survey included 754 healthy residents from Hefei during January 2011 to December 2018 reported a SFTSV-specific IgG positive rate as 20.16% (You et al., 2021). In a cohort containing 1,463 healthy individuals from 14 natural villages of Henan province during April to May 2016, the rate of seropositivity for SFTSV-specific IgG was 10.46% (Du et al., 2019). Moreover, SFTSV-specific IgM was detected in 12 individuals, and SFTSV RNA was concurrent in six of them (Du et al., 2019). Another study devoted to the miss rate of SFTS in high endemic areas of China by Chinese Center for Disease Control and Prevention, a high rate as 8.3% was reported according to the examination results of SFTSV-specific IgM and IgG (Huang et al., 2018). Combining the findings, there was a large number of asymptomatic/mild sufferers of SFTSV infection in endemic areas of China during the epidemic season. Detection of SFTSV-specific IgM/IgG and research on the risk for SFTSV spread of asymptomatic subjects are thus required, which are of special significance in the prevention and control of SFTS. In particular, it is worthy of exploring the potential factors that lead to different outcomes of SFTSV infection in same endemic areas, such as host genetic background.

It is generally known that host immunogenetics influences the outcomes of infectious disease. During SFTSV infection, viral proteins bind with cell receptors, participate in signaling regulation, and affect downstream gene transcription, which impact viral replication, immune escape, and disease outcomes. On the contrary, host relevant genes’ variations also have different effects on human susceptibility to SFTSV, disease progression and clinical outcomes. Many studies had reported family cluster SFTSV infection in China (Bao C. J. et al., 2011; Bao C. et al., 2011; Gai Z. et al., 2012; Liu et al., 2012; Chen et al., 2013; Tang et al., 2013; Sun J. et al., 2014), and high levels of SFTSV seroprevalence were showed among family members of patients with SFTS (Sun J. M. et al., 2015). A case report described that all three full sisters finally died from severe SFTSV infection, although neither interpersonal transmission nor exposure to the same suspected source of infection was found, which confirmed by SFTSV RNA sequencing (Sun J. et al., 2015). Given the self-limited nature of SFTSV infection for the majority of infected individuals, the three sisters still decreased unfortunately, suggesting their highly susceptibility to the fatal outcome of SFTSV infection, which was likely related to their common genetic background. Similarly, Shanghai Municipal Center for Disease Control and Prevention reported a case of domestic family cluster SFTSV infection, in which three members (mother, her daughter, and her son) developed severe pneumonia at early stage of infection, largely different with most SFTSV-infected cases (Zhu et al., 2017). In this case, the possible influence of genetic background on SFTSV susceptibility, clinical manifestations, and outcomes was difficult to exclude, despite the underlying interpersonal transmission through contact with blood among family members. Moreover, the infections of a variety of viruses, such as hantavirus, dengue virus, influenza virus, and hepatitis C virus, had also been verified to correlate with genetic factors (Xu-Yang et al., 2016; Kenney et al., 2017; Di Pucchio and Sekaly, 2020; Li Y. et al., 2020; Fu et al., 2021).

To date, there have been a few studies reporting the association between human genetic background and SFTSV infection. Human leukocyte antigens (HLAs) are major histocompatibility complex proteins that are located on the cell surface in human and important in antigen presentation and regulation of immune response. HLAs are highly polymorphic and polygenic. HLA alleles are highly variable in different individuals and are closely related to host immune response and the susceptibility to disease. The allele frequency distributions of *HLA-A*, *HLA-B*, and *DRB1* between two cohorts (patients with SFTS: n = 84; healthy individuals: n = 501) from Shandong Province, China, were analyzed, and it was found that haplotype A*02-B*15-DRB1*04 was positively associated with SFTSV susceptibility, whereas HLA-A*30, HLA-B*13 and haplotype A*30-B*13, A*30-B*13-DRB1*07 had negative correlation with SFTS susceptibility (Ding et al., 2016).

A Chinese case control study involving more than 2,000 subjects reported that the carriers with *platelet-derived growth factor B* (*PDGF-B*) gene *rs1800818 G* allele were more likely to get infected with SFTSV, and *rs1800818 G* allele was associated with decreased serum PDGF-BB (the name of cytokine encoded by *platelet-derived growth factor B* gene) levels in early SFTSV infection (Zhang et al., 2016). PDGF, a type of growth factors released in response to platelet aggregation and activation, controls platelet aggregation through autocrine feedback secretion (Razmi et al., 2018). PDGF family includes four subtypes, including PDGF-A, B, C, and D (Fredriksson et al., 2004; Papadopoulos and Lennartsson, 2018). PDGF molecules must form homodimers or heterodimers to be active, such as PDGF-AA, BB, AB, CC, and DD dimers (Papadopoulos and Lennartsson, 2018). In case of SFTSV infection, SFTSV adhesion resulted in the increased release of PDGF-BB from platelets, and this further led to the enhanced clearance of infected platelets by macrophages during infection, representing host active clearance of virus (Zhang et al., 2016). Yet, the individuals who carried *rs1800818 G* allele might had the deficiency in expression and release of PDGF-BB and ultimately resulted in active virus replication and high SFTSV susceptibility (Zhang et al., 2016).

TNF-α is one of the major regulators of the inflammatory response, with an important role for triggering pro-inflammatory factors, chemokines, and adhesion factor cascades. Research studies revealed that TNF-α level was significantly higher in SFTS cases versus healthy individuals, and the increase was much more evident in patients with fatal SFTS (Ding et al., 2014; Liu M. M. et al., 2017; Hu et al., 2018; Fujikawa et al., 2019). In a cohort study involving 987 hospitalized patients with SFTS and 633 asymptomatic/mild SFTS-infected subjects, five single-nucleotide polymorphisms located in the TNF-α promoter region were analyzed to evaluate their correlations with SFTS among Chinese Han population, and it was found that the presence of *rs1799964 C*/*rs361525 A* alleles and multiple haplotypes were associated with a significant reduced risk of death in patients with SFTS, and the serum TNF-α levels were related to *rs361525 A* allele (Xing et al., 2018). However, studies on the relationship between host genetic background and SFTS infection are limited, requiring more in-depth research studies.

## Other Host Factors Related to SFTS Pathogenesis

### Uridine 5′-Diphosphate-Glucose Ceramide Glucosyl-Transferase (UGCG)

It was reported that the efficient SFTSV entry into cells depended on the expression and enzymatic activity of UGCG, a Golgi transmembrane protein, which the inhibition of UGCG could hinder the post-internalization stage of SFTSV entry and thus led to impairment of viral transport and/or viral fusion with host cell membranes, although such inhibition of UGCG did not affect neither the binding nor the endocytosis of SFTSV to host cells (Drake et al., 2017).

### Sorting Nexin 11 (SNX11)

A previous study based on a whole-genome clustered regularly interspaced short palindromic repeat (CRISPR) knockout screen strategy (Liu et al., 2019b) suggested that SNX11 was also an essential host factor for SFTSV entry, because it participated in the virus intracellular transport and mediated in the synthesis and maturation of viral envelope glycoprotein in endoplasmic reticulum during SFTSV infection (Liu et al., 2019b).

### Endothelial Cells

*In vitro* SFTSV infection model indicated that the direct SFTSV infection of endothelial cells could cause the interruption of intercellular connections and significantly increased vascular permeability and the level of angiopenin-2, an inducer of inflammation (Li X. K. et al., 2018). In the meantime, S1001a8 and matrix metalloproteinase-9 levels increased, and inflammatory reaction of endothelial cells was induced, leading to the dysfunction and impaired barrier integrity of vascular endothelial cells, ultimately triggering a series of clinical lesions of SFTS infection (Li X. K. et al., 2018).

### Intercellular Adhesion Molecule 1 (ICAM-1) and Serum Amyloid A1 (SAA-1)

SFTSV can replicate in host endothelial cells. It was possible that the endothelial activation/dysfunction was a part of the pathogenesis for SFTS, and the elevated levels of serum intercellular adhesion molecule 1 and serum amyloid A1 could be used to predict severe or death outcome in patients with SFTS (Li et al., 2017). Because intercellular adhesion molecule 1 and amyloid A1 could activate of intrinsic coagulation cascade by mediating endothelial injury/activation and subsequent platelet aggregation/degranulation, thus led to hemostasis failure (Ozturk et al., 2010). All of these might be associated with occurrence of disseminated intravascular coagulation at late stage of severe and fatal SFTS cases and suggested that therapeutic interventions in endothelial dysfunction may be a strategy for SFTS treatment.

### Platelet Function and Phenotype

In a retrospective study including 1,538 confirmed patients with SFTS, it was shown that enhanced activation of cytokine/vascular endothelial cells and the disorder of coagulation function were associated with thrombocytopenia, which was involved in a variety of platelet phenotypes (Li X. K. et al., 2020). All these host responses influence SFTS outcomes in some synergistic manner, suggesting that platelet supplementation alone may to be ineffective, and novel interventions to host responses are in demand.

### Complications

A study comprising 2,096 patients with SFTS assessed the associations between clinical outcomes and multiple comorbid conditions, including hyperlipidemia, hypertension, chronic viral hepatitis, diabetes mellitus, cerebral ischemic stroke, heart diseases, chronic obstructive pulmonary disease, pulmonary tuberculosis, and cancer, and found that patients complicated with diabetes mellitus, chronic viral hepatitis, or chronic obstructive pulmonary disease tended to have fatal outcomes, probably due to the impairment of immune function (Zhang S. F. et al., 2019). Moreover, diabetes mellitus was linked to vascular complications of patients with SFTSV, which characterized by an activation of the inflammatory cascade and endothelial dysfunction (Zhang S. F. et al., 2019). In the meantime, the levels of multiple cytokines were increased evidently in patients with SFTS complicated with diabetes mellitus, which might also cause endothelial dysfunction, cytokine storm, and even a fatal outcome (Zhang S. F. et al., 2019). It was worth noting that insulin therapy significantly reduced the disease severity and might be applied as a treatment strategy (Zhang S. F. et al., 2019).

## Conclusions

In summary, SFTSV infection can lead to various clinical manifestations ranging from asymptomatic infection, mild fever to severe hemorrhage or multi-organ failure, and even death. The underlying mechanisms of SFTS pathogenesis are highly complicated and have not been completely clarified. SFTSV infection starts from viral invasion, which is supported by a subcellular environment suitable for virus replication created by virus–host interactions. During this process, the virus stimulates host innate and adaptive immune responses, with the release of vast cytokines/chemokines, and induces immunopathological damage with varying degrees. Meanwhile, host genetic factor, in turn, plays a non-negligible role in regulating functional proteins and influencing SFTSV susceptibility and disease severity; thus, further research is required. In addition, strengthened measures should be taken to educate residents in SFTS epidemic area to improve the prevention and recognition of disease. Most asymptomatic and mild SFTSV-infected individuals should be followed with surveillance to the prevention and control during the epidemic season. Because there is no SFTS specific therapy strategy, clinicians should comprehensively observe the disease phase and take timely appropriate immunotherapy by inducing, enhancing, or suppressing patients’ immune responses according to the different stages of the disease and the body’s own immune state, preferably based on evidence-based medicine. The key to improving the prognosis of patients with SFTS is to individualize treatment to maximize effectiveness and minimize risks in different patients. More in-depth studies on SFTS pathogenesis are urgently needed to screen more effective therapeutic targets and intervention strategies, as well as to provide enabling opinions to vaccine and SFTS specific drugs.

## Author Contributions

MW, WT, and MY originally drafted the manuscript and edited the manuscript. LF and JL revised the manuscript. All authors contributed to the article and approved the submitted version.

## Funding

This work was supported by Open Research Fund Program of the State Key Laboratory of State Key Laboratory of Pathogen and Biosecurity (No. SKLPBS2137), Science Foundation for Distinguished Young Scholars of Jiangsu Province (BK20190106), Key Project of Natural Science Foundation of Yunnan Province (2019FA005), and Clinical Research Center for Emerging Respiratory infectious diseases (HS2020002).

## Conflict of Interest

The authors declare that the research was conducted in the absence of any commercial or financial relationships that could be construed as a potential conflict of interest.

## Publisher’s Note

All claims expressed in this article are solely those of the authors and do not necessarily represent those of their affiliated organizations, or those of the publisher, the editors and the reviewers. Any product that may be evaluated in this article, or claim that may be made by its manufacturer, is not guaranteed or endorsed by the publisher.

## Abbreviations

SFTS, severe fever with thrombocytopenia syndrome; SFTSV, severe fever with thrombocytopenia syndrome bunyavirus; NP, nucleocapsid protein; NSs, nonstructural protein; DC-SIGN, dendritic cell–specific intercellular adhesion molecule-3-grabbing non-integrin; DCs, dendritic cells; IFN, Interferons; STAT, signal transducer and activator of transcription; IL, interleukin; TNF, tumor necrosis factor; RANTES, regulated upon activation normally T cell expressed and secreted; PDGF, platelet-derived growth factor; IP-10, IFN-γ–inducible protein; MCP, monocyte chemotactic protein; NLRP3, nucleotide-binding and oligomerization, leucine-rich repeat, and pyrin domain domain–containing protein 3; LC3, light chain 3; UGCG, uridine 5′-diphosphate-glucose ceramide glucosyl-transferase.

## References

[B1] Akagi K. Miyazaki T. Oshima K. Umemura A. Shimada S. Morita K.. (2020). Detection of Viral RNA in Diverse Body Fluids in an SFTS Patient With Encephalopathy, Gastrointestinal Bleeding and Pneumonia: A Case Report and Literature Review. BMC Infect. Dis. 20 (1), 281. doi: 10.1186/s12879-020-05012-8 32295538 PMC7160946

[B2] Aller-Garcia A. I. Castro-Mendez C. Alastruey-Izquierdo A. Marin-Martinez E. M. Breval I. Z. Couto-Caro C.. (2017). Case Series Study of Invasive Pulmonary Aspergillosis. Mycopathologia 182 (5-6), 505–515. doi: 10.1007/s11046-016-0097-1 27913978

[B3] Al-Samkari H. Berliner N. (2018). Hemophagocytic Lymphohistiocytosis. Annu. Rev. Pathol. 13, 27–49. doi: 10.1146/annurev-pathol-020117-043625 28934563

[B4] Arii J. Goto H. Suenaga T. Oyama M. Kozuka-Hata H. Imai T.. (2010). Non-Muscle Myosin IIA is a Functional Entry Receptor for Herpes Simplex Virus-1. Nature 467 (7317), 859–862. doi: 10.1038/nature09420 20944748

[B5] Bao C. J. Guo X. L. Qi X. Hu J. L. Zhou M. H. Varma J. K.. (2011). A Family Cluster of Infections by a Newly Recognized Bunyavirus in Eastern China 2007: Further Evidence of Person-to-Person Transmission. Clin. Infect. Dis. 53 (12), 1208–1214. doi: 10.1093/cid/cir732 22028437

[B6] Bao C. Qi X. Wang H. (2011). A Novel Bunyavirus in China. N. Engl. J. Med. 365 (9), 862–865. doi: 10.1056/NEJMc1106000 21879913

[B7] Bennett L. F. Liao C. Quickel M. D. Yeoh B. S. Vijay-Kumar M. Hankey-Giblin P.. (2019). Inflammation Induces Stress Erythropoiesis Through Heme-Dependent Activation of SPI-C. Sci. Signal 12 (598), eaap7336. doi: 10.1126/scisignal.aap7336 31506384 PMC6904953

[B8] Bertheloot D. Latz E. Franklin B. S. (2021). Necroptosis, Pyroptosis and Apoptosis: An Intricate Game of Cell Death. Cell Mol. Immunol. 18 (5), 1106–1121. doi: 10.1038/s41423-020-00630-3 33785842 PMC8008022

[B9] Bonneville M. Scotet E. (2006). Human Vgamma9Vdelta2 T Cells: Promising New Leads for Immunotherapy of Infections and Tumors. Curr. Opin. Immunol. 18 (5), 539–546. doi: 10.1016/j.coi.2006.07.002 16870417

[B10] Bopp N. E. Kaiser J. A. Strother A. E. Barrett A. D. T. Beasley D. W. C. Benassi V.. (2020). Baseline Mapping of Severe Fever With Thrombocytopenia Syndrome Virology, Epidemiology and Vaccine Research and Development. NPJ Vaccines 5 (1), 111. doi: 10.1038/s41541-020-00257-5 33335100 PMC7746727

[B11] Brennan B. Rezelj V. V. Elliott R. M. (2017). Mapping of Transcription Termination Within the S Segment of SFTS Phlebovirus Facilitated Generation of NSs Deletant Viruses. J. Virol. 91 (16), e00743–e00717. doi: 10.1128/JVI.00743-17 28592543 PMC5533932

[B12] Buchbinder E. Hodi F. S. (2015). Cytotoxic T Lymphocyte Antigen-4 and Immune Checkpoint Blockade. J. Clin. Invest. 125 (9), 3377–3383. doi: 10.1172/JCI80012 26325034 PMC4588295

[B13] Chaudhary V. Zhang S. Yuen K. S. Li C. Lui P. Y. Fung S. Y.. (2015). Suppression of Type I and Type III IFN Signalling by NSs Protein of Severe Fever With Thrombocytopenia Syndrome Virus Through Inhibition of STAT1 Phosphorylation and Activation. J. Gen. Virol. 96 (11), 3204–3211. doi: 10.1099/jgv.0.000280 26353965

[B14] Chen H. Hu K. Zou J. Xiao J. (2013). A Cluster of Cases of Human-to-Human Transmission Caused by Severe Fever With Thrombocytopenia Syndrome Bunyavirus. Int. J. Infect. Dis. 17 (3), e206–e208. doi: 10.1016/j.ijid.2012.11.006 23218674

[B15] Chen X. Ye H. Li S. Jiao B. Wu J. Zeng P.. (2017). Severe Fever With Thrombocytopenia Syndrome Virus Inhibits Exogenous Type I IFN Signaling Pathway Through its NSs *In Vitro* . PLoS One 12 (2), e0172744. doi: 10.1371/journal.pone.0172744 28234991 PMC5325526

[B16] Choi Y. Bowman J. W. Jung J. U. (2018). Autophagy During Viral Infection - a Double-Edged Sword. Nat. Rev. Microbiol. 16 (6), 341–354. doi: 10.1038/s41579-018-0003-6 29556036 PMC6907743

[B17] Choi Y. Jiang Z. Shin W. J. Jung J. U. (2020). Severe Fever With Thrombocytopenia Syndrome Virus NSs Interacts With TRIM21 To Activate the P62-Keap1-Nrf2 Pathway. J. Virol. 94 (6), e01684–e01619. doi: 10.1128/JVI.01684-19 31852783 PMC7158713

[B18] Choi Y. Park S. J. Sun Y. Yoo J. S. Pudupakam R. S. Foo S. S.. (2019). Severe Fever With Thrombocytopenia Syndrome Phlebovirus Non-Structural Protein Activates TPL2 Signalling Pathway for Viral Immunopathogenesis. Nat. Microbiol. 4 (3), 429–437. doi: 10.1038/s41564-018-0329-x 30617349 PMC6548314

[B19] Cui N. Bao X. L. Yang Z. D. Lu Q. B. Hu C. Y. Wang L. Y.. (2014). Clinical Progression and Predictors of Death in Patients With Severe Fever With Thrombocytopenia Syndrome in China. J. Clin. Virol. 59 (1), 12–17. doi: 10.1016/j.jcv.2013.10.024 24257109

[B20] Cui N. Liu R. Lu Q. B. Wang L. Y. Qin S. L. Yang Z. D.. (2015). Severe Fever With Thrombocytopenia Syndrome Bunyavirus-Related Human Encephalitis. J. Infect. 70 (1), 52–59. doi: 10.1016/j.jinf.2014.08.001 25135231

[B21] Dendrou C. A. Petersen J. Rossjohn J. Fugger L. (2018). HLA Variation and Disease. Nat. Rev. Immunol. 18 (5), 325–339. doi: 10.1038/nri.2017.143 29292391

[B22] Deng B. Zhang S. Geng Y. Zhang Y. Wang Y. Yao W.. (2012). Cytokine and Chemokine Levels in Patients With Severe Fever With Thrombocytopenia Syndrome Virus. PLoS One 7 (7), e41365. doi: 10.1371/journal.pone.0041365 22911786 PMC3404083

[B23] Deng B. Zhou B. Zhang S. Zhu Y. Han L. Geng Y.. (2013). Clinical Features and Factors Associated With Severity and Fatality Among Patients With Severe Fever With Thrombocytopenia Syndrome Bunyavirus Infection in Northeast China. PLoS One 8 (11), e80802. doi: 10.1371/journal.pone.0080802 24236203 PMC3827460

[B24] Ding Y. P. Liang M. F. Ye J. B. Liu Q. H. Xiong C. H. Long B.. (2014). Prognostic Value of Clinical and Immunological Markers in Acute Phase of SFTS Virus Infection. Clin. Microbiol. Infect. 20 (11), O870–O878. doi: 10.1111/1469-0691.12636 24684627

[B25] Ding S. J. Zhang Y. Zhang X. M. Jiang X. L. Pang B. Song Y. H.. (2016). Correlation Between HLA-A, B and DRB1 Alleles and Severe Fever With Thrombocytopenia Syndrome. PLoS Negl. Trop. Dis. 10 (10), e0005076. doi: 10.1371/journal.pntd.0005076 27760141 PMC5070855

[B26] Di Pucchio T. Sekaly R. P. (2020). HLA Polymorphism and Tapasin Independence Influence Outcomes of HIV and Dengue Virus Infection. Proc. Natl. Acad. Sci. U. S. A. 117 (50), 31570–31572. doi: 10.1073/pnas.2020109117 33239443 PMC7749352

[B27] Drake M. J. Brennan B. Briley K. Jr. Bart S. M. Sherman E. Szemiel A. M.. (2017). A Role for Glycolipid Biosynthesis in Severe Fever With Thrombocytopenia Syndrome Virus Entry. PLoS Pathog. 13 (4), e1006316. doi: 10.1371/journal.ppat.1006316 28388693 PMC5397019

[B28] Du Y. Cheng N. Li Y. Wang H. You A. Su J.. (2019). Seroprevalance of Antibodies Specific for Severe Fever With Thrombocytopenia Syndrome Virus and the Discovery of Asymptomatic Infections in Henan Province, China. PLoS Negl. Trop. Dis. 13 (11), e0007242. doi: 10.1371/journal.pntd.0007242 31765376 PMC6901261

[B29] Fajgenbaum D. C. June C. H. (2020). Cytokine Storm. N. Engl. J. Med. 383 (23), 2255–2273. doi: 10.1056/NEJMra2026131 33264547 PMC7727315

[B30] Fredriksson L. Li H. Eriksson U. (2004). The PDGF Family: Four Gene Products Form Five Dimeric Isoforms. Cytokine Growth Factor. Rev. 15 (4), 197–204. doi: 10.1016/j.cytogfr.2004.03.007 15207811

[B31] Fu Z. Cai W. Shao J. Xue H. Ge Z. Fan H.. (2021). Genetic Variants in TNFSF4 and TNFSF8 Are Associated With the Risk of HCV Infection Among Chinese High-Risk Population. Front. Genet. 12. doi: 10.3389/fgene.2021.630310 PMC8027328 33841497

[B32] Fujikawa K. Koga T. Honda T. Uchida T. Okamoto M. Endo Y.. (2019). Serial Analysis of Cytokine and Chemokine Profiles and Viral Load in Severe Fever With Thrombocytopenia Syndrome: Case Report and Review of Literature. Med. (Baltimore) 98 (42), e17571. doi: 10.1097/MD.0000000000017571 PMC6824633 31626125

[B33] Gai Z. Liang M. Zhang Y. Zhang S. Jin C. Wang S. W.. (2012). Person-To-Person Transmission of Severe Fever With Thrombocytopenia Syndrome Bunyavirus Through Blood Contact. Clin. Infect. Dis. 54 (2), 249–252. doi: 10.1093/cid/cir776 22095565 PMC3245727

[B34] Gai Z. T. Zhang Y. Liang M. F. Jin C. Zhang S. Zhu C. B.. (2012). Clinical Progress and Risk Factors for Death in Severe Fever With Thrombocytopenia Syndrome Patients. J. Infect. Dis. 206 (7), 1095–1102. doi: 10.1093/infdis/jis472 22850122

[B35] Garcia-Vallejo J. J. van Kooyk Y. (2013). The Physiological Role of DC-SIGN: A Tale of Mice and Men. Trends Immunol. 34 (10), 482–486. doi: 10.1016/j.it.2013.03.001 23608151

[B36] Giri M. S. Nebozyhn M. Raymond A. Gekonge B. Hancock A. Creer S.. (2009). Circulating Monocytes in HIV-1-Infected Viremic Subjects Exhibit an Antiapoptosis Gene Signature and Virus- and Host-Mediated Apoptosis Resistance. J. Immunol. 182 (7), 4459–4470. doi: 10.4049/jimmunol.0801450 19299747 PMC2776064

[B37] Gong L. Zhang L. Wu J. Lu S. Lyu Y. Zhu M.. (2021). Clinical Progress and Risk Factors for Death From Severe Fever With Thrombocytopenia Syndrome: A Multihospital Retrospective Investigation in Anhui, China. Am. J. Trop. Med. Hyg. 104 (4), 1425–1431. doi: 10.4269/ajtmh.20-0270 33591933 PMC8045620

[B38] Halldorsson S. Behrens A. J. Harlos K. Huiskonen J. T. Elliott R. M. Crispin M.. (2016). Structure of a Phleboviral Envelope Glycoprotein Reveals a Consolidated Model of Membrane Fusion. Proc. Natl. Acad. Sci. U. S. A. 113 (26), 7154–7159. doi: 10.1073/pnas.1603827113 27325770 PMC4932967

[B39] He Z. Wang B. Li Y. Hu K. Yi Z. Ma H.. (2021). Changes in Peripheral Blood Cytokines in Patients With Severe Fever With Thrombocytopenia Syndrome. J. Med. Virol. 93 (8), 4704–4713. doi: 10.1002/jmv.26877 33590892 PMC8360139

[B40] Hiraki T. Yoshimitsu M. Suzuki T. Goto Y. Higashi M. Yokoyama S.. (2014). Two Autopsy Cases of Severe Fever With Thrombocytopenia Syndrome (SFTS) in Japan: A Pathognomonic Histological Feature and Unique Complication of SFTS. Pathol. Int. 64 (11), 569–575. doi: 10.1111/pin.12207 25329676 PMC4282027

[B41] Hofmann H. Li X. Zhang X. Liu W. Kuhl A. Kaup F.. (2013). Severe Fever With Thrombocytopenia Virus Glycoproteins are Targeted by Neutralizing Antibodies and can Use DC-SIGN as a Receptor for pH-Dependent Entry Into Human and Animal Cell Lines. J. Virol. 87 (8), 4384–4394. doi: 10.1128/JVI.02628-12 23388721 PMC3624395

[B42] Hong Y. Bai M. Qi X. Li C. Liang M. Li D.. (2019). Suppression of the IFN-Alpha and -Beta Induction Through Sequestering IRF7 Into Viral Inclusion Bodies by Nonstructural Protein NSs in Severe Fever With Thrombocytopenia Syndrome Bunyavirus Infection. J. Immunol. 202 (3), 841–856. doi: 10.4049/jimmunol.1800576 30598516

[B43] Huang X. Wang S. Wang X. Lyu Y. Jiang M. Chen D.. (2018). Estimation of the Incidence of Severe Fever With Thrombocytopenia Syndrome in High Endemic Areas in China: An Inpatient-Based Retrospective Study. BMC Infect. Dis. 18 (1), 66. doi: 10.1186/s12879-018-2970-7 29402229 PMC5800001

[B44] Hu L. F. Wu T. Wang B. Wei Y. Y. Kong Q. X. Ye Y.. (2018). The Regulation of Seventeen Inflammatory Mediators Are Associated With Patient Outcomes in Severe Fever With Thrombocytopenia Syndrome. Sci. Rep. 8 (1), 159. doi: 10.1038/s41598-017-18616-z 29317732 PMC5760584

[B45] Imataki O. Uemura M. Masugata H. (2019). Severe Rhabdomyolysis Associated With Severe Fever With Thrombocytopenia Syndrome in a Married Couple: A Case Report. BMC Infect. Dis. 19 (1), 885. doi: 10.1186/s12879-019-4535-9 31651242 PMC6813050

[B46] Imre G. (2020). Cell Death Signalling in Virus Infection. Cell Signal 76, 109772. doi: 10.1016/j.cellsig.2020.109772 32931899 PMC7486881

[B47] Italiani P. Boraschi D. (2014). From Monocytes to M1/M2 Macrophages: Phenotypical vs. Functional Differentiation. Front. Immunol. 5. doi: 10.3389/fimmu.2014.00514 PMC4201108 25368618

[B48] Iwao K. Kawaguchi T. Kimura M. Iwao C. Rikitake M. Aizawa A.. (2021). Severe Fever With Thrombocytopenia Syndrome Accompanied by Invasive Pulmonary Aspergillosis: An Autopsy Case. Viruses 13 (6), 1086. doi: 10.3390/v13061086 34200385 PMC8226712

[B49] Jin C. Liang M. Ning J. Gu W. Jiang H. Wu W.. (2012). Pathogenesis of Emerging Severe Fever With Thrombocytopenia Syndrome Virus in C57/BL6 Mouse Model. Proc. Natl. Acad. Sci. U. S. A. 109 (25), 10053–10058. doi: 10.1073/pnas.1120246109 22665769 PMC3382536

[B50] Jung I. Y. Ahn K. Kim J. Choi J. Y. Kim H. Y. Uh Y.. (2019a). Higher Fatality for Severe Fever With Thrombocytopenia Syndrome Complicated by Hemophagocytic Lymphohistiocytosis. Yonsei. Med. J. 60 (6), 592–596. doi: 10.3349/ymj.2019.60.6.592 31124344 PMC6536390

[B51] Jung I. Y. Choi W. Kim J. Wang E. Park S. W. Lee W. J.. (2019b). Nosocomial Person-to-Person Transmission of Severe Fever With Thrombocytopenia Syndrome. Clin. Microbiol. Infect. 25 (5), 633.e1–633.e4. doi: 10.1016/j.cmi.2019.01.006 30677496

[B52] Kaneko M. Shikata H. Matsukage S. Maruta M. Shinomiya H. Suzuki T.. (2018). A Patient With Severe Fever With Thrombocytopenia Syndrome and Hemophagocytic Lymphohistiocytosis-Associated Involvement of the Central Nervous System. J. Infect. Chemother. 24 (4), 292–297. doi: 10.1016/j.jiac.2017.10.016 29138019

[B53] Kawaguchi T. Matsuda M. Takajo I. Kawano A. Kariya Y. Kubo K.. (2016). Severe Fever With Thrombocytopenia Syndrome With Myocardial Dysfunction and Encephalopathy: A Case Report. J. Infect. Chemother. 22 (9), 633–637. doi: 10.1016/j.jiac.2016.01.022 26943978

[B54] Keller M. D. Torres V. J. Cadwell K. (2020). Autophagy and Microbial Pathogenesis. Cell Death Differ. 27 (3), 872–886. doi: 10.1038/s41418-019-0481-8 31896796 PMC7205878

[B55] Kenney A. D. Dowdle J. A. Bozzacco L. McMichael T. M. St Gelais C. Panfil A. R.. (2017). Human Genetic Determinants of Viral Diseases. Annu. Rev. Genet. 51, 241–263. doi: 10.1146/annurev-genet-120116-023425 28853921 PMC6038703

[B56] Khalil J. Yamada S. Tsukamoto Y. Abe H. Shimojima M. Kato H.. (2020). The Non-Structural Protein NSs of SFTSV Causes Cytokine Storm Through the Hyper-Activation of NF-kappaB. Mol. Cell Biol. 41 (3), e00542–e00520. doi: 10.1128/MCB.00542-20 PMC8088271 33288641

[B57] Kim K. H. Lee M. J. Ko M. K. Lee E. Y. Yi J. (2018). Severe Fever With Thrombocytopenia Syndrome Patients With Hemophagocytic Lymphohistiocytosis Retrospectively Identified in Korea 2008-2013. J. Korean Med. Sci. 33 (50), e319. doi: 10.3346/jkms.2018.33.e319 30534031 PMC6281956

[B58] Klei T. R. Meinderts S. M. van den Berg T. K. van Bruggen R. (2017). From the Cradle to the Grave: The Role of Macrophages in Erythropoiesis and Erythrophagocytosis. Front. Immunol. 8. doi: 10.3389/fimmu.2017.00073 PMC5288342 28210260

[B59] Kobayashi Y. Kato H. Yamagishi T. Shimada T. Matsui T. Yoshikawa T.. (2020). Severe Fever With Thrombocytopenia Syndrome, Japan 2013-2017. Emerg. Infect. Dis. 26 (4), 692–699. doi: 10.3201/eid2604.191011 32186502 PMC7101122

[B60] Kubes P. Jenne C. (2018). Immune Responses in the Liver. Annu. Rev. Immunol. 36, 247–277. doi: 10.1146/annurev-immunol-051116-052415 29328785

[B61] Kuhn J. H. Adkins S. Alioto D. Alkhovsky S. V. Amarasinghe G. K. Anthony S. J.. (2020). 2020 Taxonomic Update for Phylum Negarnaviricota (Riboviria: Orthornavirae), Including the Large Orders Bunyavirales and Mononegavirales. Arch. Virol. 165 (12), 3023–3072. doi: 10.1007/s00705-020-04731-2 32888050 PMC7606449

[B62] Kwon J. S. Kim M. C. Kim J. Y. Jeon N. Y. Ryu B. H. Hong J.. (2018). Kinetics of Viral Load and Cytokines in Severe Fever With Thrombocytopenia Syndrome. J. Clin. Virol. 101, 57–62. doi: 10.1016/j.jcv.2018.01.017 29427908 PMC7106421

[B63] Lampugnani M. G. Elisabetta D. (2007). The Control of Endothelial Cell Functions by Adherens Junctions. Novartis Foundation Symposium 283, 4–13. doi: 10.1002/9780470319413.ch2 18300410

[B64] Lampugnani M. G. Orsenigo F. Gagliani M. C. Tacchetti C. Dejana E. (2006). Vascular Endothelial Cadherin Controls VEGFR-2 Internalization and Signaling From Intracellular Compartments. J. Cell Biol. 174 (4), 593–604. doi: 10.1083/jcb.200602080 16893970 PMC2064264

[B65] Lee J. Jeong G. Lim J. H. Kim H. Park S. W. Lee W. J.. (2016). Severe Fever With Thrombocytopenia Syndrome Presenting With Hemophagocytic Lymphohistiocytosis. Infect. Chemother. 48 (4), 338–341. doi: 10.3947/ic.2016.48.4.338 27883371 PMC5204015

[B66] Lev S. Gottesman T. Sahaf Levin G. Lederfein D. Berkov E. Diker D.. (2021). Observational Cohort Study of IP-10’s Potential as a Biomarker to Aid in Inflammation Regulation Within a Clinical Decision Support Protocol for Patients With Severe COVID-19. PLoS One 16 (1), e0245296. doi: 10.1371/journal.pone.0245296 33434221 PMC7802954

[B67] Liang S. Wu Y. S. Li D. Y. Tang J. X. Liu H. F. (2021). Autophagy in Viral Infection and Pathogenesis. Front. Cell Dev. Biol. 9. doi: 10.3389/fcell.2021.766142 PMC8554085 34722550

[B68] Li Y. Chen X. Y. Gu W. M. Qian H. M. Tian Y. Tang J.. (2020). A Meta-Analysis of Tumor Necrosis Factor (TNF) Gene Polymorphism and Susceptibility to Influenza A (H1n1). Comput. Biol. Chem. 89, 107385. doi: 10.1016/j.compbiolchem.2020.107385 33032038

[B69] Li X. K. Dai K. Yang Z. D. Yuan C. Cui N. Zhang S. F.. (2020). Correlation Between Thrombocytopenia and Host Response in Severe Fever With Thrombocytopenia Syndrome. PLoS Negl. Trop. Dis. 14 (10), e0008801. doi: 10.1371/journal.pntd.0008801 33119592 PMC7595704

[B70] Li J. Han Y. Xing Y. Li S. Kong L. Zhang Y.. (2014). Concurrent Measurement of Dynamic Changes in Viral Load, Serum Enzymes, T Cell Subsets, and Cytokines in Patients With Severe Fever With Thrombocytopenia Syndrome. PLoS One 9 (3), e91679. doi: 10.1371/journal.pone.0091679 24658451 PMC3962368

[B71] Li Z. Hu J. Bao C. Gao C. Zhang N. Cardona C. J.. (2022). Activation of the NLRP3 Inflammasome and Elevation of Interleukin-1beta Secretion in Infection by Sever Fever With Thrombocytopenia Syndrome Virus. Sci. Rep. 12 (1), 2573. doi: 10.1038/s41598-022-06229-0 35173184 PMC8850576

[B72] Li Y. Li H. Wang H. Pan H. Zhao H. Jin H.. (2019). The Proportion, Origin and Pro-Inflammation Roles of Low Density Neutrophils in SFTS Disease. BMC Infect. Dis. 19 (1), 109. doi: 10.1186/s12879-019-3701-4 30717709 PMC6360754

[B73] Li S. Li Y. Wang Q. Yu X. Liu M. Xie H.. (2018). Multiple Organ Involvement in Severe Fever With Thrombocytopenia Syndrome: An Immunohistochemical Finding in a Fatal Case. Virol. J. 15 (1), 97. doi: 10.1186/s12985-018-1006-7 29848330 PMC5977472

[B74] Li J. Li S. Yang L. Cao P. Lu J. (2021). Severe Fever With Thrombocytopenia Syndrome Virus: A Highly Lethal Bunyavirus. Crit. Rev. Microbiol. 47 (1), 112–125. doi: 10.1080/1040841X.2020.1847037 33245676

[B75] Li S. Li H. Zhang Y. L. Xin Q. L. Guan Z. Q. Chen X.. (2020). SFTSV Infection Induces BAK/BAX-Dependent Mitochondrial DNA Release to Trigger NLRP3 Inflammasome Activation. Cell Rep. 30 (13), 4370–4385.e7. doi: 10.1016/j.celrep.2020.02.105 32234474

[B76] Li X. Lu Q. Chen W. Xu W. Liu R. Zhang S.. (2018). Arginine Deficiency Is Involved in Thrombocytopenia and Immunosuppression in Severe Fever With Thrombocytopenia Syndrome. Sci. Trans. Med. 10 (459), eaat4162. doi: 10.1126/scitranslmed.aat4162 30232226

[B77] Li H. Lu Q.-B. Xing B. Zhang S.-F. Liu K. Du J.. (2018). Epidemiological and Clinical Features of Laboratory-Diagnosed Severe Fever With Thrombocytopenia Syndrome in China 2011–17: A Prospective Observational Study. Lancet Infect. Dis. 18 (10), 1127–1137. doi: 10.1016/s1473-3099(18)30293-7 30054190

[B78] Liu S. Chai C. Wang C. Amer S. Lv H. He H.. (2014). Systematic Review of Severe Fever With Thrombocytopenia Syndrome: Virology, Epidemiology, and Clinical Characteristics. Rev. Med. Virol. 24 (2), 90–102. doi: 10.1002/rmv.1776 24310908 PMC4237196

[B79] Liu Q. He B. Huang S. Y. Wei F. Zhu X. Q. (2014). Severe Fever With Thrombocytopenia Syndrome, an Emerging Tick-Borne Zoonosis. Lancet Infect. Dis. 14 (8), 763–772. doi: 10.1016/s1473-3099(14)70718-2 24837566

[B80] Liu M. M. Lei X. Y. Yu X. J. (2016). Meta-Analysis of the Clinical and Laboratory Parameters of SFTS Patients in China. Virol. J. 13 (1), 198. doi: 10.1186/s12985-016-0661-9 27899121 PMC5129669

[B81] Liu M. M. Lei X. Y. Yu H. Zhang J. Z. Yu X. J. (2017). Correlation of Cytokine Level With the Severity of Severe Fever With Thrombocytopenia Syndrome. Virol. J. 14 (1), 6. doi: 10.1186/s12985-016-0677-1 28086978 PMC5237221

[B82] Liu Y. Li Q. Hu W. Wu J. Wang Y. Mei L.. (2012). Person-To-Person Transmission of Severe Fever With Thrombocytopenia Syndrome Virus. Vector Borne Zoonotic Dis. 12 (2), 156–160. doi: 10.1089/vbz.2011.0758 21955213

[B83] Liu T. Li J. Liu Y. Qu Y. Li A. Li C.. (2019b). SNX11 Identified as an Essential Host Factor for SFTS Virus Infection by CRISPR Knockout Screening. Virol. Sin. 34 (5), 508–520. doi: 10.1007/s12250-019-00141-0 31215001 PMC6814687

[B84] Liu S. Liu H. Kang J. Xu L. Zhang K. Li X.. (2020). The Severe Fever With Thrombocytopenia Syndrome Virus NSs Protein Interacts With CDK1 To Induce G2 Cell Cycle Arrest and Positively Regulate Viral Replication. J. Virol. 94 (6), e01575-19. doi: 10.1128/JVI.01575-19 PMC7158732 31852787

[B85] Liu J. Wang L. Feng Z. Geng D. Sun Y. Yuan G. (2017). Dynamic Changes of Laboratory Parameters and Peripheral Blood Lymphocyte Subsets in Severe Fever With Thrombocytopenia Syndrome Patients. Int. J. Infect. Dis. 58, 45–51. doi: 10.1016/j.ijid.2017.02.017 28249810

[B86] Liu J. Xu M. Tang B. Hu L. Deng F. Wang H.. (2019a). Single-Particle Tracking Reveals the Sequential Entry Process of the Bunyavirus Severe Fever With Thrombocytopenia Syndrome Virus. Small 15 (6), e1803788. doi: 10.1002/smll.201803788 30589216

[B87] Li M. Xiong Y. Li M. Zhang W. Liu J. Zhang Y.. (2020). Depletion But Activation of CD56(dim)CD16(+) NK Cells in Acute Infection With Severe Fever With Thrombocytopenia Syndrome Virus. Virol. Sin. 35 (5), 588–598. doi: 10.1007/s12250-020-00224-3 32430872 PMC7736421

[B88] Li X. K. Yang Z. D. Du J. Xing B. Cui N. Zhang P. H.. (2017). Endothelial Activation and Dysfunction in Severe Fever With Thrombocytopenia Syndrome. PLoS Negl. Trop. Dis. 11 (8), e0005746. doi: 10.1371/journal.pntd.0005746 28806760 PMC5581191

[B89] Li M. M. Zhang W. J. Liu J. Li M. Y. Zhang Y. F. Xiong Y.. (2018a). Dynamic Changes in the Immunological Characteristics of T Lymphocytes in Surviving Patients With Severe Fever With Thrombocytopenia Syndrome (SFTS). Int. J. Infect. Dis. 70, 72–80. doi: 10.1016/j.ijid.2018.03.010 29550447

[B90] Li M. M. Zhang W. J. Weng X. F. Li M. Y. Liu J. Xiong Y.. (2018b). CD4 T Cell Loss and Th2 and Th17 Bias Are Associated With the Severity of Severe Fever With Thrombocytopenia Syndrome (SFTS). Clin. Immunol. 195, 8–17. doi: 10.1016/j.clim.2018.07.009 30036637 PMC7185468

[B91] Li X. K. Zhang S. F. Xu W. Xing B. Lu Q. B. Zhang P. H.. (2018). Vascular Endothelial Injury in Severe Fever With Thrombocytopenia Syndrome Caused by the Novel Bunyavirus. Virology 520, 11–20. doi: 10.1016/j.virol.2018.05.001 29754008

[B92] Lu Q. B. Cui N. Hu J. G. Chen W. W. Xu W. Li H.. (2015). Characterization of Immunological Responses in Patients With Severe Fever With Thrombocytopenia Syndrome: A Cohort Study in China. Vaccine 33 (10), 1250–1255. doi: 10.1016/j.vaccine.2015.01.051 25645176

[B93] Matsuno K. Orba Y. Maede-White K. Scott D. Feldmann F. Liang M.. (2017). Animal Models of Emerging Tick-Borne Phleboviruses: Determining Target Cells in a Lethal Model of SFTSV Infection. Front. Microbiol. 8. doi: 10.3389/fmicb.2017.00104 PMC5276813 28194148

[B94] Miao D. Liu M. J. Wang Y. X. Ren X. Lu Q. B. Zhao G. P.. (2021). Epidemiology and Ecology of Severe Fever With Thrombocytopenia Syndrome in China 2010-2018. Clin. Infect. Dis. 73 (11), e3851–e3858. doi: 10.1093/cid/ciaa1561 33068430 PMC8664468

[B95] Min Y. Q. Ning Y. J. Wang H. Deng F. (2020). A RIG-I-Like Receptor Directs Antiviral Responses to a Bunyavirus and is Antagonized by Virus-Induced Blockade of TRIM25-Mediated Ubiquitination. J. Biol. Chem. 295 (28), 9691–9711. doi: 10.1074/jbc.RA120.013973 32471869 PMC7363118

[B96] Miyamoto S. Ito T. Terada S. Eguchi T. Furubeppu H. Kawamura H.. (2019). Fulminant Myocarditis Associated With Severe Fever With Thrombocytopenia Syndrome: A Case Report. BMC Infect. Dis. 19 (1), 266. doi: 10.1186/s12879-019-3904-8 30885147 PMC6423866

[B97] Mizushima N. Levine B. (2020). Autophagy in Human Diseases. N. Engl. J. Med. 383 (16), 1564–1576. doi: 10.1056/NEJMra2022774 33053285

[B98] Moriyama M. Igarashi M. Koshiba T. Irie T. Takada A. Ichinohea T. (2018). Two Conserved Amino Acids Within the NSs of Severe Fever With Thrombocytopenia Syndrome Phlebovirus Are Essential for Anti-Interferon Activity. J. Virol. 92 (19), e00706-00718. doi: 10.1128/JVI.00706-18 30021900 PMC6146818

[B99] Nakamura S. Azuma M. Maruhashi T. Sogabe K. Sumitani R. Uemura M.. (2018). Steroid Pulse Therapy in Patients With Encephalopathy Associated With Severe Fever With Thrombocytopenia Syndrome. J. Infect. Chemother. 24 (5), 389–392. doi: 10.1016/j.jiac.2017.11.004 29428565

[B100] Nakamura S. Iwanaga N. Hara S. Shimada S. Kashima Y. Hayasaka D.. (2019). Viral Load and Inflammatory Cytokine Dynamics Associated With the Prognosis of Severe Fever With Thrombocytopenia Syndrome Virus Infection: An Autopsy Case. J. Infect. Chemother. 25 (6), 480–484. doi: 10.1016/j.jiac.2019.01.013 30824300

[B101] Nakano A. Ogawa H. Nakanishi Y. Fujita H. Mahara F. Shiogama K.. (2017). Hemophagocytic Lymphohistiocytosis in a Fatal Case of Severe Fever With Thrombocytopenia Syndrome. Intern. Med. 56 (12), 1597–1602. doi: 10.2169/internalmedicine.56.6904 28626191 PMC5505921

[B102] Ning Y. J. Feng K. Min Y. Q. Cao W. C. Wang M. Deng F.. (2015). Disruption of Type I Interferon Signaling by the Nonstructural Protein of Severe Fever With Thrombocytopenia Syndrome Virus *via* the Hijacking of STAT2 and STAT1 Into Inclusion Bodies. J. Virol. 89 (8), 4227–4236. doi: 10.1128/JVI.00154-15 25631085 PMC4442386

[B103] Ning Y. J. Mo Q. Feng K. Min Y. Q. Li M. Hou D.. (2019). Interferon-Gamma-Directed Inhibition of a Novel High-Pathogenic Phlebovirus and Viral Antagonism of the Antiviral Signaling by Targeting Stat1. Front. Immunol. 10. doi: 10.3389/fimmu.2019.01182 PMC6546826 31191546

[B104] Ning Y. J. Wang M. Deng M. Shen S. Liu W. Cao W. C.. (2014). Viral Suppression of Innate Immunity *via* Spatial Isolation of TBK1/IKKepsilon From Mitochondrial Antiviral Platform. J. Mol. Cell Biol. 6 (4), 324–337. doi: 10.1093/jmcb/mju015 24706939 PMC7107466

[B105] Niu G. Li J. Liang M. Jiang X. Jiang M. Yin H.. (2013). Severe Fever With Thrombocytopenia Syndrome Virus Among Domesticated Animals, China. Emerg. Infect. Dis. 19 (5), 756–763. doi: 10.3201/eid1905.120245 23648209 PMC3647489

[B106] Oh H. S. Kim M. Lee J. O. Kim H. Kim E. S. Park K. U.. (2016). Hemophagocytic Lymphohistiocytosis Associated With SFTS Virus Infection: A Case Report With Literature Review. Med. (Baltimore) 95 (31), e4476. doi: 10.1097/MD.0000000000004476 PMC4979843 27495089

[B107] Ozturk B. Kuscu F. Tutuncu E. Sencan I. Gurbuz Y. Tuzun H. (2010). Evaluation of the Association of Serum Levels of Hyaluronic Acid, sICAM-1, sVCAM-1, and VEGF-A With Mortality and Prognosis in Patients With Crimean-Congo Hemorrhagic Fever. J. Clin. Virol. 47 (2), 115–119. doi: 10.1016/j.jcv.2009.10.015 20005156

[B108] Palis J. (2014). Primitive and Definitive Erythropoiesis in Mammals. Front. Physiol. 5. doi: 10.3389/fphys.2014.00003 PMC3904103 24478716

[B109] Papadopoulos N. Lennartsson J. (2018). The PDGF/PDGFR Pathway as a Drug Target. Mol. Aspects Med. 62, 75–88. doi: 10.1016/j.mam.2017.11.007 29137923

[B110] Park S. Y. Kwon J. S. Kim J. Y. Kim S. M. Jang Y. R. Kim M. C.. (2018). Severe Fever With Thrombocytopenia Syndrome-Associated Encephalopathy/Encephalitis. Clin. Microbiol. Infect. 24 (4), 432.e1–432.e4. doi: 10.1016/j.cmi.2017.09.002 28899841

[B111] Park A. Park S. J. Jung K. L. Kim S. M. Kim E. H. Kim Y. I.. (2021). Molecular Signatures of Inflammatory Profile and B-Cell Function in Patients With Severe Fever With Thrombocytopenia Syndrome. Am. Soc. FOR Microbiol. 12 (1), e02583–e02520. doi: 10.1128/mBio.02583-20 PMC8545090 33593977

[B112] Paulson R. F. Hariharan S. Little J. A. (2020a). Stress Erythropoiesis: Definitions and Models for its Study. Exp. Hematol. 89, 43–54.e42. doi: 10.1016/j.exphem.2020.07.011 32750404 PMC7508762

[B113] Paulson R. F. Ruan B. Hao S. Chen Y. (2020b). Stress Erythropoiesis Is a Key Inflammatory Response. Cells 9 (3), 634. doi: 10.3390/cells9030634 PMC7140438 32155728

[B114] Peng C. Wang H. Zhang W. Zheng X. Tong Q. Jie S.. (2016). Decreased Monocyte Subsets and TLR4-Mediated Functions in Patients With Acute Severe Fever With Thrombocytopenia Syndrome (SFTS). Int. J. Infect. Dis. 43, 37–42. doi: 10.1016/j.ijid.2015.12.009 26701820

[B115] Plegge T. Hofmann-Winkler H. Spiegel M. Pohlmann S. (2016). Evidence That Processing of the Severe Fever With Thrombocytopenia Syndrome Virus Gn/Gc Polyprotein Is Critical for Viral Infectivity and Requires an Internal Gc Signal Peptide. PLoS One 11 (11), e0166013. doi: 10.1371/journal.pone.0166013 27855227 PMC5113920

[B116] Qu B. Qi X. Wu X. Liang M. Li C. Cardona C. J.. (2012). Suppression of the Interferon and NF-kappaB Responses by Severe Fever With Thrombocytopenia Syndrome Virus. J. Virol. 86 (16), 8388–8401. doi: 10.1128/JVI.00612-12 22623799 PMC3421730

[B117] Razmi N. Baradaran B. Hejazi M. Hasanzadeh M. Mosafer J. Mokhtarzadeh A.. (2018). Recent Advances on Aptamer-Based Biosensors to Detection of Platelet-Derived Growth Factor. Biosens. Bioelectron. 113, 58–71. doi: 10.1016/j.bios.2018.04.048 29729560

[B118] Runwal G. Stamatakou E. Siddiqi F. H. Puri C. Zhu Y. Rubinsztein D. C. (2019). LC3-Positive Structures are Prominent in Autophagy-Deficient Cells. Sci. Rep. 9 (1), 10147. doi: 10.1038/s41598-019-46657-z 31300716 PMC6625982

[B119] Saijo M. (2018). Pathophysiology of Severe Fever With Thrombocytopenia Syndrome and Development of Specific Antiviral Therapy. J. Infect. Chemother. 24 (10), 773–781. doi: 10.1016/j.jiac.2018.07.009 30098914

[B120] Saraiva M. Vieira P. O'Garra A. (2020). Biology and Therapeutic Potential of Interleukin-10. J. Exp. Med. 217 (1), e20190418. doi: 10.1084/jem.20190418 31611251 PMC7037253

[B121] Seu K. G. Papoin J. Fessler R. Hom J. Huang G. Mohandas N.. (2017). Unraveling Macrophage Heterogeneity in Erythroblastic Islands. Front. Immunol. 8. doi: 10.3389/fimmu.2017.01140 PMC5611421 28979259

[B122] Sharma D. Kanneganti T. D. (2016). The Cell Biology of Inflammasomes: Mechanisms of Inflammasome Activation and Regulation. J. Cell Biol. 213 (6), 617–629. doi: 10.1083/jcb.201602089 27325789 PMC4915194

[B123] Song P. Zheng N. Liu Y. Tian C. Wu X. Ma X.. (2018). Deficient Humoral Responses and Disrupted B-Cell Immunity Are Associated With Fatal SFTSV Infection. Nat. Commun. 9 (1), 3328. doi: 10.1038/s41467-018-05746-9 30127439 PMC6102208

[B124] Song P. Zheng N. Zhang L. Liu Y. Chen T. Bao C.. (2017). Downregulation of Interferon-Beta and Inhibition of TLR3 Expression Are Associated With Fatal Outcome of Severe Fever With Thrombocytopenia Syndrome. Sci. Rep. 7 (1), 6532. doi: 10.1038/s41598-017-06921-6 28747721 PMC5529500

[B125] Speth C. Hagleitner M. Ott H. W. Wurzner R. Lass-Florl C. Rambach G. (2013). Aspergillus Fumigatus Activates Thrombocytes by Secretion of Soluble Compounds. J. Infect. Dis. 207 (5), 823–833. doi: 10.1093/infdis/jis743 23225903

[B126] Spiegel M. Plegge T. Pohlmann S. (2016). The Role of Phlebovirus Glycoproteins in Viral Entry, Assembly and Release. Viruses 8 (7), 202. doi: 10.3390/v8070202 PMC4974537 27455305

[B127] Sun J. Chai C. Lv H. Lin J. Wang C. Chen E.. (2014). Epidemiological Characteristics of Severe Fever With Thrombocytopenia Syndrome in Zhejiang Province, China. Int. J. Infect. Dis. 25, 180–185. doi: 10.1016/j.ijid.2014.02.022 24947422

[B128] Sun L. Hu Y. Niyonsaba A. Tong Q. Lu L. Li H.. (2014). Detection and Evaluation of Immunofunction of Patients With Severe Fever With Thrombocytopenia Syndrome. Clin. Exp. Med. 14 (4), 389–395. doi: 10.1007/s10238-013-0259-0 24068614 PMC7101760

[B129] Sun Y. Jin C. Zhan F. Wang X. Liang M. Zhang Q.. (2012). Host Cytokine Storm Is Associated With Disease Severity of Severe Fever With Thrombocytopenia Syndrome. J. Infect. Dis. 206 (7), 1085–1094. doi: 10.1093/infdis/jis452 22904342

[B130] Sun Q. Jin C. Zhu L. Liang M. Li C. Cardona C. J.. (2015). Host Responses and Regulation by NFkappaB Signaling in the Liver and Liver Epithelial Cells Infected With A Novel Tick-Borne Bunyavirus. Sci. Rep. 5, 11816. doi: 10.1038/srep11816 26134299 PMC4488873

[B131] Sun Y. Liu M. M. Lei X. Y. Yu X. J. (2018). SFTS Phlebovirus Promotes LC3-II Accumulation and Nonstructural Protein of SFTS Phlebovirus Co-Localizes With Autophagy Proteins. Sci. Rep. 8 (1), 5287. doi: 10.1038/s41598-018-23610-0 29588492 PMC5869591

[B132] Sun Y. Qi Y. Liu C. Gao W. Chen P. Fu L.. (2014). Nonmuscle Myosin Heavy Chain IIA is a Critical Factor Contributing to the Efficiency of Early Infection of Severe Fever With Thrombocytopenia Syndrome Virus. J. Virol. 88 (1), 237–248. doi: 10.1128/JVI.02141-13 24155382 PMC3911693

[B133] Sun Q. Qi X. Zhang Y. Wu X. Liang M. Li C.. (2016). Synaptogyrin-2 Promotes Replication of a Novel Tick-Borne Bunyavirus Through Interacting With Viral Nonstructural Protein NSs. J. Biol. Chem. 291 (31), 16138–16149. doi: 10.1074/jbc.M116.715599 27226560 PMC4965563

[B134] Sun J. Tang Y. Ling F. Chang Y. Ye X. Shi W.. (2015). Genetic Susceptibility Is One of the Determinants for Severe Fever With Thrombocytopenia Syndrome Virus Infection and Fatal Outcome: An Epidemiological Investigation. PLoS One 10 (7), e0132968. doi: 10.1371/journal.pone.0132968 26207638 PMC4514768

[B135] Sun J. M. Zhang Y. J. Gong Z. Y. Zhang L. Lv H. K. Lin J. F.. (2015). Seroprevalence of Severe Fever With Thrombocytopenia Syndrome Virus in Southeastern China and Analysis of Risk Factors. Epidemiol. Infect. 143 (4), 851–856. doi: 10.1017/S0950268814001319 24866248 PMC4411641

[B136] Suzuki T. Sato Y. Sano K. Arashiro T. Katano H. Nakajima N.. (2020). Severe Fever With Thrombocytopenia Syndrome Virus Targets B Cells in Lethal Human Infections. J. Clin. Invest. 130 (2), 799–812. doi: 10.1172/JCI129171 31904586 PMC6994144

[B137] Swanson K. V. Deng M. Ting J. P. (2019). The NLRP3 Inflammasome: Molecular Activation and Regulation to Therapeutics. Nat. Rev. Immunol. 19 (8), 477–489. doi: 10.1038/s41577-019-0165-0 31036962 PMC7807242

[B138] Takahashi T. Maeda K. Suzuki T. Ishido A. Shigeoka T. Tominaga T.. (2014). The First Identification and Retrospective Study of Severe Fever With Thrombocytopenia Syndrome in Japan. J. Infect. Dis. 209 (6), 816–827. doi: 10.1093/infdis/jit603 24231186 PMC7107388

[B139] Tang X. Wu W. Wang H. Du Y. Liu L. Kang K.. (2013). Human-To-Human Transmission of Severe Fever With Thrombocytopenia Syndrome Bunyavirus Through Contact With Infectious Blood. J. Infect. Dis. 207 (5), 736–739. doi: 10.1093/infdis/jis748 23225899

[B140] Uehara N. Yano T. Ishihara A. Saijou M. Suzuki T. (2016). Fatal Severe Fever With Thrombocytopenia Syndrome: An Autopsy Case Report. Intern. Med. 55 (7), 831–838. doi: 10.2169/internalmedicine.55.5262 27041174

[B141] Vicente-Manzanares M. Ma X. Adelstein R. S. Horwitz A. R. (2009). Non-Muscle Myosin II Takes Centre Stage in Cell Adhesion and Migration. Nat. Rev. Mol. Cell Biol. 10 (11), 778–790. doi: 10.1038/nrm2786 19851336 PMC2834236

[B142] Wang G. Chang H. Jia B. Liu Y. Huang R. Wu W.. (2019). Nucleocapsid Protein-Specific IgM Antibody Responses in the Disease Progression of Severe Fever With Thrombocytopenia Syndrome. Ticks Tick. Borne Dis. 10 (3), 639–646. doi: 10.1016/j.ttbdis.2019.02.003 30824322

[B143] Wang N. Liang H. Zen K. (2014). Molecular Mechanisms That Influence the Macrophage M1-M2 Polarization Balance. Front. Immunol. 5. doi: 10.3389/fimmu.2014.00614 PMC4246889 25506346

[B144] Weng Y. Chen N. Han Y. Xing Y. Li J. (2014). Clinical and Laboratory Characteristics of Severe Fever With Thrombocytopenia Syndrome in Chinese Patients. Braz. J. Infect. Dis. 18 (1), 88–91. doi: 10.1016/j.bjid.2013.05.011 24076112 PMC9425216

[B145] Westover J. B. Hickerson B. T. Van Wettere A. J. Hurst B. L. Kurz J. P. Dagley A.. (2019). Vascular Leak and Hypercytokinemia Associated With Severe Fever With Thrombocytopenia Syndrome Virus Infection in Mice. Pathogens 8 (4), 158. doi: 10.3390/pathogens8040158 PMC6963364 31546590

[B146] Xing B. Li X. K. Zhang S. F. Lu Q. B. Du J. Zhang P. H.. (2018). Polymorphisms and Haplotypes in the Promoter of the TNF-Alpha Gene Are Associated With Disease Severity of Severe Fever With Thrombocytopenia Syndrome in Chinese Han Population. PLoS Negl. Trop. Dis. 12 (6), e0006547. doi: 10.1371/journal.pntd.0006547 29939989 PMC6034906

[B147] Xu S. Jiang N. Nawaz W. Liu B. Zhang F. Liu Y.. (2021). Infection of Humanized Mice With a Novel Phlebovirus Presented Pathogenic Features of Severe Fever With Thrombocytopenia Syndrome. PLoS Pathog. 17 (5), e1009587. doi: 10.1371/journal.ppat.1009587 33974679 PMC8139491

[B148] Xu W. Li X. K. Lu Q. B. Yang Z. D. Du J. Xing B.. (2017). Association Between Peripheral Gammadelta T Cell Subsets and Disease Progression of Severe Fever With Thrombocytopenia Syndrome Virus Infection. Pathog. Dis. 75 (7), ftx086. doi: 10.1093/femspd/ftx086 28859400

[B149] Xu-Yang Z. Pei-Yu B. Chuan-Tao Y. Wei Y. Hong-Wei M. Kang T.. (2016). Interferon-Induced Transmembrane Protein 3 Inhibits Hantaan Virus Infection, and Its Single Nucleotide Polymorphism Rs12252 Influences the Severity of Hemorrhagic Fever With Renal Syndrome. Front. Immunol. 7. doi: 10.3389/fimmu.2016.00535 PMC5206578 28096800

[B150] Yajima T. (2011). Viral Myocarditis: Potential Defense Mechanisms Within the Cardiomyocyte Against Virus Infection. Future Microbiol. 6 (5), 551–566. doi: 10.2217/fmb.11.40 21585262 PMC3131135

[B151] Yamaoka S. Weisend C. Ebihara H. (2020). Identifying Target Cells for a Tick-Borne Virus That Causes Fatal Hemorrhagic Fever. J. Clin. Invest. 130 (2), 598–600. doi: 10.1172/JCI134512 31904585 PMC6994110

[B152] Yan J. M. Zhang W. K. Yan L. N. Jiao Y. J. Zhou C. M. Yu X. J. (2021). Bunyavirus SFTSV Exploits Autophagic Flux for Viral Assembly and Egress. Autophagy, 1–14. doi: 10.1080/15548627.2021.1994296 PMC9298452 34747299

[B153] Yoo J. R. Heo S. T. Park D. Kim H. Fukuma A. Fukushi S.. (2016). Family Cluster Analysis of Severe Fever With Thrombocytopenia Syndrome Virus Infection in Korea. Am. J. Trop. Med. Hyg. 95 (6), 1351–1357. doi: 10.4269/ajtmh.16-0527 27928083 PMC5154449

[B154] Yoo J. R. Kim T. J. Heo S. T. Hwang K. A. Oh H. Ha T.. (2021). IL-6 and IL-10 Levels, Rather Than Viral Load and Neutralizing Antibody Titers, Determine the Fate of Patients With Severe Fever With Thrombocytopenia Syndrome Virus Infection in South Korea. Front. Immunol. 12. doi: 10.3389/fimmu.2021.711847 PMC8416084 34484214

[B155] Yoshikawa T. Fukushi S. Tani H. Fukuma A. Taniguchi S. Toda S.. (2014). Sensitive and Specific PCR Systems for Detection of Both Chinese and Japanese Severe Fever With Thrombocytopenia Syndrome Virus Strains and Prediction of Patient Survival Based on Viral Load. J. Clin. Microbiol. 52 (9), 3325–3333. doi: 10.1128/JCM.00742-14 24989600 PMC4313158

[B156] You E. Wang L. Zhang L. Wu J. Zhao K. Huang F. (2021). Epidemiological Characteristics of Severe Fever With Thrombocytopenia Syndrome in Hefei of Anhui Province: A Population-Based Surveillance Study From 2011 to 2018. Eur. J. Clin. Microbiol. Infect. Dis. 40 (5), 929–939. doi: 10.1007/s10096-020-04098-x 33188497

[B157] Yu X. Liang M. Zhang S. Liu Y. Li J. Sun Y.. (2011). Fever With Thrombocytopenia Associated With a Novel Bunyavirus in China. N. Engl. J. Med. 364 (16), 1523–1532. doi: 10.1056/NEJMoa1010095 21410387 PMC3113718

[B158] Zhang L. Fu Y. Wang H. Guan Y. Zhu W. Guo M.. (2019). Severe Fever With Thrombocytopenia Syndrome Virus-Induced Macrophage Differentiation Is Regulated by miR-146. Front. Immunol. 10. doi: 10.3389/fimmu.2019.01095 PMC6529556 31156641

[B159] Zhang X. Guo C. Lu Q. Liu W. Hu J. Cao W.. (2016). The Platelet Derived Growth Factor-B Polymorphism is Associated With Risk of Severe Fever With Thrombocytopenia Syndrome in Chinese Individuals. Oncotarget 7 (22), 33340–33349. doi: 10.18632/oncotarget.9043 27147565 PMC5078099

[B160] Zhang Y. Z. He Y. W. Dai Y. A. Xiong Y. Zheng H. Zhou D. J.. (2012). Hemorrhagic Fever Caused by a Novel Bunyavirus in China: Pathogenesis and Correlates of Fatal Outcome. Clin. Infect. Dis. 54 (4), 527–533. doi: 10.1093/cid/cir804 22144540

[B161] Zhang W. Li M. Xiong S. Wang H. Xiong Y. Li M.. (2017). Decreased Myeloid Dendritic Cells Indicate a Poor Prognosis in Patients With Severe Fever With Thrombocytopenia Syndrome. Int. J. Infect. Dis. 54, 113–120. doi: 10.1016/j.ijid.2016.11.418 27915109

[B162] Zhang F. Ren S. Zuo Y. (2014). DC-SIGN, DC-SIGNR and LSECtin: C-Type Lectins for Infection. Int. Rev. Immunol. 33 (1), 54–66. doi: 10.3109/08830185.2013.834897 24156700

[B163] Zhang S. F. Yang Z. D. Huang M. L. Wang Z. B. Hu Y. Y. Miao D.. (2019). Preexisting Chronic Conditions for Fatal Outcome Among SFTS Patients: An Observational Cohort Study. PLoS Negl. Trop. Dis. 13 (5), e0007434. doi: 10.1371/journal.pntd.0007434 31136581 PMC6555536

[B164] Zhang J. Yan X. Li Y. Gao R. Wang P. Mo W. (2018). Reactive Plasmacytosis Mimicking Multiple Myeloma Associated With SFTS Virus Infection: A Report of Two Cases and Literature Review. BMC Infect. Dis. 18 (1), 528. doi: 10.1186/s12879-018-3431-z 30348099 PMC6198377

[B165] Zhou X. Jiang W. Liu Z. Liu S. Liang X. (2017). Virus Infection and Death Receptor-Mediated Apoptosis. Viruses 9 (11), 316. doi: 10.3390/v9110316 PMC5707523 29077026

[B166] Zhou H. Sun Y. Wang Y. Liu M. Liu C. Wang W.. (2013). The Nucleoprotein of Severe Fever With Thrombocytopenia Syndrome Virus Processes a Stable Hexameric Ring to Facilitate RNA Encapsidation. Protein Cell 4 (6), 445–455. doi: 10.1007/s13238-013-3901-4 23702688 PMC4875558

[B167] Zhu Y. Wu H. Gao J. Zhou X. Zhu R. Zhang C.. (2017). Two Confirmed Cases of Severe Fever With Thrombocytopenia Syndrome With Pneumonia: Implication for a Family Cluster in East China. BMC Infect. Dis. 17 (1), 537. doi: 10.1186/s12879-017-2645-9 28774267 PMC5541732

